# Fermiology-driven goniopolar transverse thermoelectricity in kagome metals

**DOI:** 10.1038/s41563-026-02678-4

**Published:** 2026-07-31

**Authors:** Haihua Hu, Yiwei Ju, Erjian Cheng, Xiaolong Feng, Fei Sun, Rui Lou, Walter Schnelle, Ralf Koban, Honghui Wang, Alexander Fedorov, Oleksandr Suvorov, Anupam Jana, Jun Fujii, Ivana Vobornik, Denis V. Vyalikh, Bernd Büchner, Bin He, Xiaoqing Pan, Claudia Felser

**Affiliations:** 1 Max Planck Institute for Chemical Physics of Solids, Dresden, Germany; 2 Department of Materials Science and Engineering, University of California, Irvine, CA USA; 3 Leibniz Institute for Solid State and Materials Research, IFW Dresden, Dresden, Germany; 4 Helmholtz-Zentrum Berlin für Materialien und Energie, Berlin, Germany; 5 Joint Laboratory ‘Functional Quantum Materials’ at BESSY II, Berlin, Germany; 6 CNR-IOM Istituto Officina dei Materiali, Area Science Park, Trieste, Italy; 7 International Centre for Theoretical Physics (ICTP), Trieste, Italy; 8 Donostia International Physics Center (DIPC), Donostia-San Sebastián, Spain; 9 IKERBASQUE, Basque Foundation for Science, Bilbao, Spain; 10 Institute of Solid State and Materials Physics, TU Dresden, Dresden, Germany; 11 Irvine Materials Research Institute (IMRI), University of California, Irvine, CA USA

**Keywords:** Thermoelectrics, Electronic properties and materials

## Abstract

The exotic geometry of the kagome lattice drives emergent quantum states and advances energy technologies; however, the anomalous Nernst effect (ANE)-based magnetic systems are fundamentally limited by low thermopowers (<6μ V K^−1^) and stray-field interference. Here we propose goniopolarity (axis-dependent carrier polarity) to achieve high zero-field transverse thermoelectric responses in kagome systems. By exploiting flat-band- and van Hove singularity-driven electronic states, we uncover exceptionally large goniopolar thermoelectric responses in LuCo_6_Ge_6_, including a transverse thermopower of 18.4 μV K^−1^ and a transverse Peltier conductivity of 105 A m^−1^ K^−1^ at room temperature and zero field. The synergy of flat bands with high electrical conductivity yields values an order of magnitude greater than those achieved in conventional ANE-based systems. Our findings establish goniopolar kagome metals as promising candidates for thermoelectrics.

## Abstract (web summary)

Axis-dependent carrier polarity, in combination with flat-band and van Hove singularity states, enable transverse thermopower of 18.4 μV K^−1^ and transverse Peltier conductivity of 105 A m^−1^ K^−1^ at room temperature and zero field in LuCo_6_Ge_6_.

## Main

The kagome lattice, a two-dimensional network of corner-sharing triangles, serves as an archetypal platform for probing correlated electron physics and emergent quantum states^1^. In kagome materials, the structural geometry naturally hosts Dirac fermions, flat bands and van Hove singularities (VHSs) in their electronic structure, and a hierarchy of exotic quantum-ordered phases has been uncovered^2–4^. Beyond their pivotal role in quantum matter exploration, kagome materials exhibit transformative potential for energy technologies, particularly in thermoelectrics^5,6^. Thermoelectricity is a solid-state energy conversion mechanism that enables the direct interconversion of thermal and electrical energy^7–9^. It addresses key challenges in waste heat recovery and advanced thermal management, offering a pathway towards carbon-neutral industrial operations^10^.

Recent studies have highlighted the potential of transverse thermoelectric effects for energy harvesting due to their ability to efficiently cover curved heat sources across large areas in a flexible manner^8,11–13^. However, high-efficiency transverse thermoelectrics typically require an external magnetic field for operation, which inevitably induces substantial electromagnetic interference^14,15^. This interference poses a challenge for integrating transverse thermoelectrics with other electronic devices. Currently, strategies leveraging the anomalous Nernst effect (ANE) and goniopolarity have been developed to achieve zero-field transverse thermoelectrics^12,16–18^. The ANE originates from time-reversal symmetry breaking and the Berry curvature in magnetic topological systems^19–21^. The ANE thermopower remains an order of magnitude lower than the Seebeck coefficient^22^, indicating that the merit still falls short of commercial applications. Because strong stray fields and the inherent difficulty of harnessing intrinsic Berry curvature in magnetic materials severely limit their development, it is essential to explore alternative mechanisms for zero-field transverse energy conversion.

Fortunately, goniopolarity offers a promising approach to achieving high-efficiency transverse thermoelectric conversion in nonmagnetic systems under zero magnetic field^18,23–25^. Goniopolarity, also known as axis-dependent carrier polarity, enables opposite carrier polarities (p-type or n-type) in a single material along different crystallographic axes. Notably, it can contribute to higher transverse thermopowers compared with ANE. Moreover, goniopolarity allows broad tunability of thermoelectric properties through optimization of carrier concentration and mobility, constituting a critical advantage for adaptive application requirements^26^. Despite its promise, current design frameworks and material options remain limited, leaving plenty of scope for further exploration. However, the pioneering work by Mahan and Sofo proposed that thermoelectricity would be enhanced with a δ-shape transport distribution^27^; the kagome lattice hosting flat bands and VHSs at varied fillings exactly fulfils this criterion, which is featured by the enhanced electronic density of states (DOS), suggesting substantial potential for large thermopowers. Despite the characteristic flat bands and VHSs in kagome systems, whether kagome-specific electronic structures can generate goniopolarity remains unknown. Here, motivated by the intriguing electronic structure, we propose a fermiology-driven goniopolar transport framework involving two distinct mechanisms: a flat-band-enabled two-band mechanism and a VHS-driven single-band mechanism. Guided by theoretical models, we experimentally realize high zero-field transverse thermoelectric responses in LuCo_6_Ge_6_ and its siblings Lu(Ho)Cr_6_Ge_6_. Our results establish kagome materials as a natural platform for goniopolar transport and provide a conceptual route for engineering transverse thermoelectricity through electronic-structure design.

## Fermiology models of goniopolar kagome lattices

**Fig. 1:**
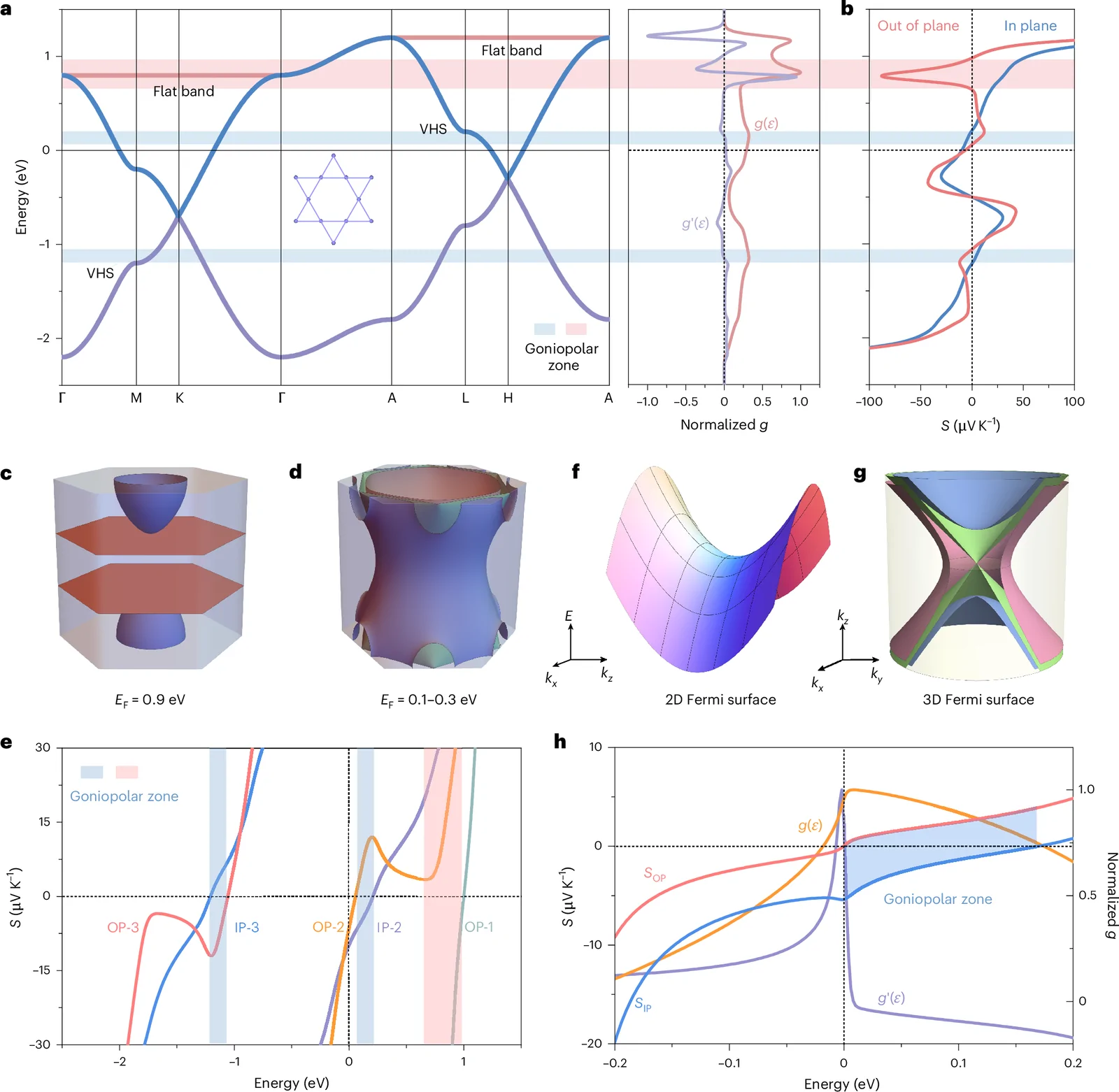
Goniopolarity in kagome lattice. **a**, Band structure of single-orbital kagome models featuring exclusively in-plane and out-of-plane nearest hoppings. The DOS *g*(*ε*) and its derivative *g*′(*ε*) are shown on the right side, where *ε* is the chemical potential. The parameters are set as *a* = 1, *c* = 1, *t*_i_ = −0.5 and *t*_o_ = −0.2, where *a* and *c* are the in-plane and out-of-plane lattice constants, respectively, and *t*_i_ and *t*_o_ denote the in-plane and out-of-plane hopping. Inset: the typical kagome lattice. **b**, Corresponding in-plane *S*_IP_ (blue) and out-of-plane *S*_OP_ (red) Seebeck coefficients. The single-band and two-band goniopolar zones, marked in blue and red shading, respectively, show opposite signs for in-plane (IP) and out-of-plane (OP) Seebeck response. These zones are positioned near flat bands and VHSs. **c**,**d**, Typical Fermi surface at Fermi level *E*_F_ = 0.9 eV (**c**), illustrating the exact flat bands (red) and VHS (blue), whereas *E*_F_ = 0.1 eV (blue), 0.2 eV (green), 0.3 eV (red) (**d**), showing the Fermi surface around the VHS. **e**, Band-resolved Seebeck coefficients with shaded areas for single-band (blue) and two-band (red) goniopolar zones. **f**, Two-dimensional Fermi surface for VHS with a saddle point. **g**, Three-dimensional Fermi surface for VHS at *E*_F_ = 0 (green), +*Δ* (red) and −*Δ* (blue). *Δ* denotes the energy deviation from zero energy. The parameters for VHS are set as: *c*_i_ = 1 and |*k*_i_| < 0.5, where *c*_i_ is a model parameter and *k*_i_ is the momentum. **h**, Goniopolarity in VHS, including the in-plane and out-of-plane Seebeck response. Normalized DOS *g*(*ε*) and its derivative *g*′(*ε*) are also presented. A strong *t*_o_ case is shown in Supplementary Fig. 1. Source data

The electronic structure of a two-dimensional (2D) kagome lattice with the nearest-neighbour hopping exhibits two defining features: perfectly flat bands and logarithmically divergent VHSs^28^. In contrast, extending to a three-dimensional (3D) kagome lattice fundamentally alters these properties. Incorporation of the nearest-neighbour out-of-plane hopping imparts dispersion to the formerly flat bands along the *k*_*z*_ direction, enabling out-of-plane charge transport. Concurrently, VHSs lose their divergent characters in three dimensions, leading to suppressed electron instabilities^29–31^. Figure 1a shows the characteristic band structure and DOS of a 3D kagome lattice, considering only the nearest-neighbour in-plane and out-of-plane hoppings. The DOS reveals two key signatures: (1) prominent peaks arising from the dispersive flat bands and (2) smaller features attributed to non-divergent VHSs. On the basis of these electronic distinctions, we propose two fermiology-related mechanisms driving goniopolar transport in kagome lattices: (1) flat-band-enabled two-band mechanism and (2) single-band mechanism enabled by VHS-driven asynchronous particle–hole asymmetry.

In 3D flat bands, transport primarily occurs in the out-of-plane direction, because the in-plane transport behaviours are strongly suppressed by vanishing group velocity or diverging effective mass (Supplementary Fig. 2). Consequently, an additional dispersive band dominates the in-plane transport, inducing anisotropy in this two-band system. When these bands comprise one electron-like and one hole-like band, the system exhibits goniopolar transport. As shown in Fig. 1c (Fermi level *E*_F_ = 0.9 eV), the Fermi surface reveals an in-plane flat band for out-of-plane transport and an isotropic hole pocket for in-plane transport. This results in goniopolar Seebeck responses within the red shaded zone (Fig. 1b,e). The enhanced DOS of flat bands contributes substantially to the Seebeck response peak.

VHSs show distinct particle–hole symmetry characteristics across dimensions. In 2D systems, conventional VHSs maintain strict particle–hole symmetry^28^. Conversely, 3D VHSs are inherently particle–hole asymmetric. Such asymmetry directly leads to the observable thermoelectric responses, which can be described by **S** = **σ**^−1^**α** $={-\frac{{{\rm\pi }}^{2}}{3}\frac{{\kappa }_{\mathrm{B}}^{2}T}{|e|}(\frac{{\rm{d}}\mathrm{ln}g}{{\rm{d}}\varepsilon }+\frac{{\rm{d}}\mathrm{ln}{v}^{2}}{{\rm{d}}\varepsilon }+\frac{{\rm{d}}\mathrm{ln}\tau }{{\rm{d}}\varepsilon })|}_{\varepsilon =E}$, where **σ** is the electrical conductivity, **α** is the Peltier conductivity, *k*_B_ is the Boltzmann constant, *e* is the elementary charge, *T* is the temperature, *g* is the density of states, *v* is the group velocity and *τ* is the relaxation time. The contributions can be ascribed to three components: the DOS, the kinetic energy and the relaxation time, quantifying the competition between electron and hole energy flux. When the electron and hole contributions are kept in balance, the thermal responses vanish, which means that the imbalance determines both the magnitude and sign of the response. Thus, thermoelectric measurements serve as direct probes of particle–hole symmetry at finite temperatures. Transport anisotropy of VHSs generates asynchronous particle–hole asymmetry along the in-plane and out-of-plane directions, allowing for single-band goniopolarity. The single-band goniopolar zones near VHSs are highlighted in Fig. 1b,e (blue-shaded zone). The corresponding Fermi surface around the VHS is illustrated in Fig. 1d.

To elucidate the single-band goniopolarity, we analyse the Hamiltonian $H={c}_{1}{k}_{x}^{2}+{c}_{2}{k}_{y}^{2}-{c}_{3}{k}_{z}^{2}$. Figure 1f,g depicts the Fermi surfaces near the saddle point in two and three dimensions, respectively: hyperbolic along *k*_*z*_, yet circular within the plane. The Fermi surface consists of two separate parabolic sheets below the saddle point, which merge into a single hyperbolic surface above it, emphasizing the transition occurring at the saddle point. When the Fermi level is below the saddle point, the energy flux increases as the Fermi level increases in both the in-plane and out-of-plane directions, contributed by the increasing DOS, indicating a negative Seebeck response. When the Fermi level is above the saddle point, the derivative of the DOS exhibits a sudden sign reversal, leading to negative contributions in both the in-plane and out-of-plane directions. However, the kinetic energy is still positive and increasing in the in-plane direction while it is always negative in the out-of-plane direction due to the opposite masses, leading to the goniopolar response (Fig. 1h and Supplementary Fig. 3). The theoretical analysis suggests that the sign reversal of the out-of-plane response can be captured by the evolution of boundaries of the Fermi surface due to the depletion of high-kinetic-energy states. When the Fermi level is high enough, the energy flux decreases in both the in-plane and out-of-plane directions due to the decreasing DOS, indicating a positive Seebeck response. Therefore, the goniopolar behaviour in single-band systems arises from asynchronous particle–hole asymmetry caused by anisotropic kinetic distributions of opposite masses^18,32,33^.

## Characterization of kagome single crystals

Guided by fermiology models, we selected three 166-type kagome metals (LuCo_6_Ge_6_, LuCr_6_Ge_6_ and HoCr_6_Ge_6_) to experimentally investigate the goniopolar effect. Three paramagnetic metals (>10 K) were crystallized in a typical kagome lattice structure (Supplementary Figs. 4–6) with a space group of *P*6/*mmm* (No. 191). The clear diffraction spots observed in the Laue diffraction patterns of these metals serve as evidence for a perfect single-crystal structure. Furthermore, the heat capacity measurements of the three kagome metals reveal no discernible phase or magnetic transitions between 10 K and 300 K.

**Fig. 2:**
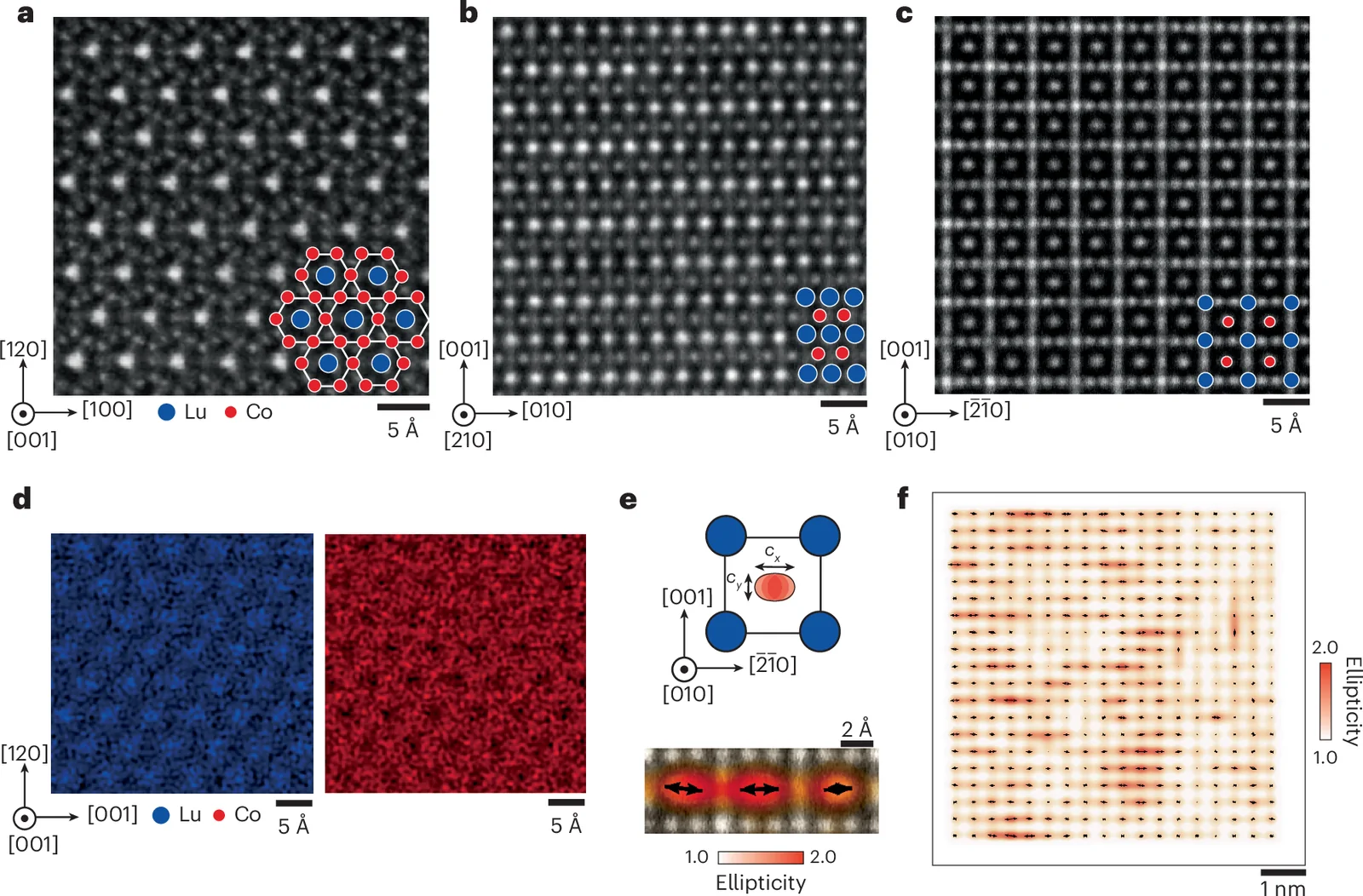
Atomic-resolution STEM results of the single-crystalline LuCo_6_Ge_6_ kagome metal. **a**–**c**, HAADF-STEM images of LuCo_6_Ge_6_ viewed along the [001] (**a**), [210] (**b**) and [010] (**c**) zone axes. The Lu and Co sites are highlighted with blue and red circles, respectively. **d**, EDS mappings of Lu and Co elements. The field of view is same as in **a**. **e**, Top: schematic graph of the definition of ellipticity in Co atomic columns when viewed along the [010] zone axis. The *c*_*x*_ and *c*_*y*_ represent the standard deviation along two orthogonal directions when fitting the 2D Gaussian peak (see Methods for more details). Bottom: HAADF-STEM image overlapped with measured ellipticity. The colour represents the ellipticity and the arrow represents the elongated direction. **f**, Ellipticity of Co atomic columns measured from 8 × 8 nm^2^.

Scanning transmission electron microscopy (STEM) was used to investigate the atomic structures. High-angle annular dark-field STEM (HAADF-STEM) images (Supplementary Figs. 7–9) reveal well-ordered atomic arrangements of LuCo_6_Ge_6_, LuCr_6_Ge_6_ and HoCr_6_Ge_6_, with distinct kagome lattices formed by Co or Cr atoms. HAADF-STEM images of LuCo_6_Ge_6_ along the [001], [210] and [010] zone axes are presented in Fig. 2a–c, where Lu and Co atoms are highlighted by blue and red circles, respectively. Figure 2d shows the energy-dispersive spectroscopy (EDS) maps of Lu and Co elements from the same field of view as Fig. 2a. These results indicate the high crystal quality and uniform elemental distribution of the LuCo_6_Ge_6_ sample. In addition, the Co atomic columns show elongation along in-plane directions when viewed along the [010] axis, as evidenced by measuring ellipticity. As defined in Fig. 2e, the ellipticity can be determined by the ratio between *c*_*x*_ and *c*_*y*_, where the *c*_*x*_ and *c*_*y*_ represent the standard deviation along two orthogonal directions when fitting the 2D Gaussian peak. Figure 2f shows the measured ellipticity of the Co site in LuCo_6_Ge_6_ when viewed along the [010] zone axis, demonstrating elongation along the in-plane direction. This elongation suggests a slight in-plane disorder shift of Co atoms from their ideal lattice positions, leading to a minor distortion of the in-plane lattice. It is noteworthy that slight in-plane distortion might cause in-plane carrier scattering, reducing carrier relaxation time and thereby inducing anisotropic resistivities in LuCo_6_Ge_6_ (ref. ^34^; Supplementary Fig. 10). However, the impact of disorder shift on the kagome structure is negligible, as indicated by the powder X-ray diffraction data (Supplementary Fig. 4).

## Goniopolarity and transverse thermoelectric effect

**Fig. 3:**
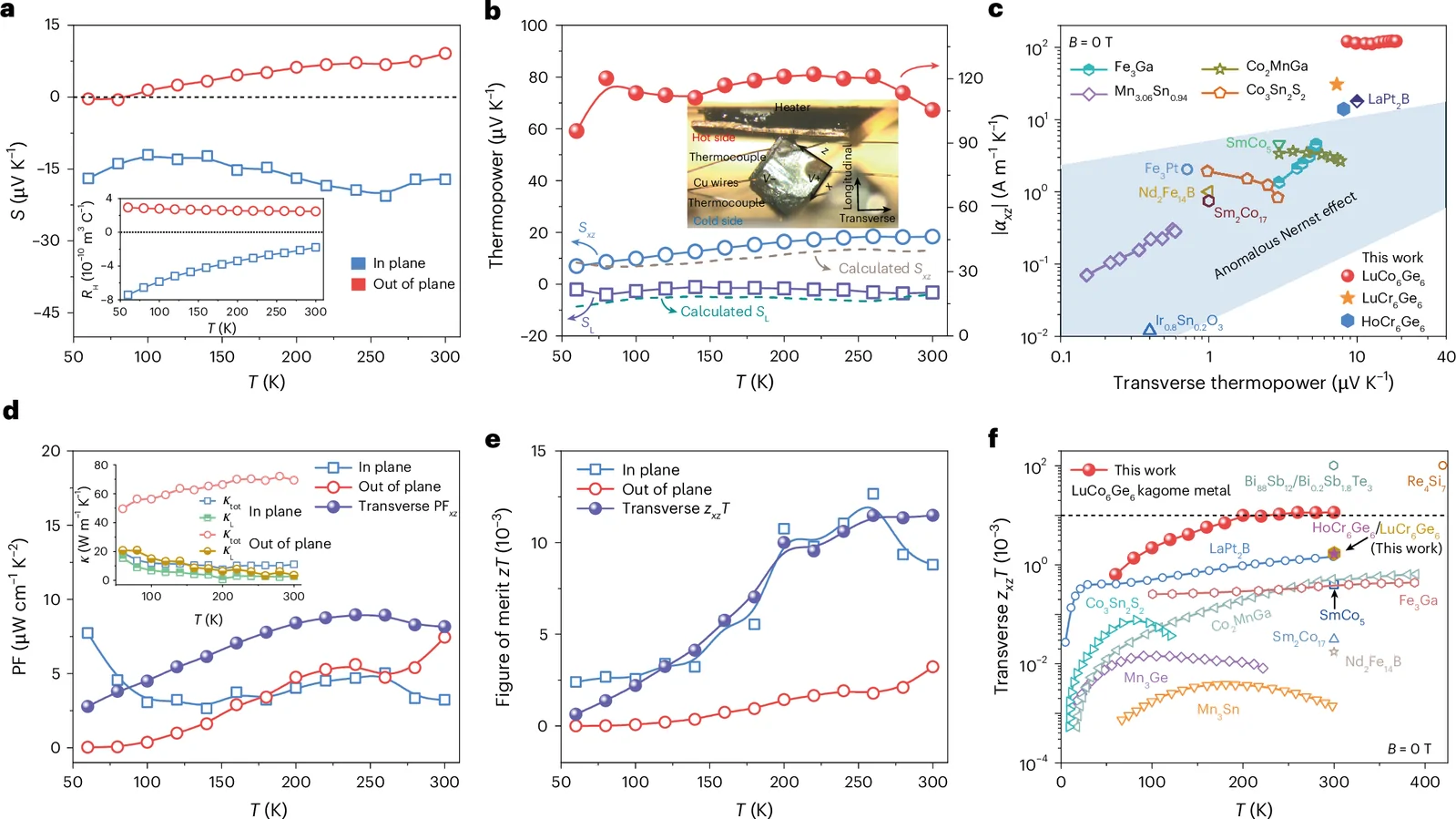
Thermoelectric transport properties of the single-crystalline LuCo_6_Ge_6_ kagome metal. **a**, Temperature-dependent in-plane (*S*_IP_) and out-of-plane (*S*_OP_) Seebeck coefficients of the LuCo_6_Ge_6_ single crystal. Inset: corresponding Hall coefficients (*R*_H_) as a function of temperature. **b**, Longitudinal thermopower *S*_L_, transverse goniopolar thermopower *S*_*xz*_ and transverse Peltier conductivity |*α*_*xz*_| versus temperature. Theoretical *S*_L_ and *S*_*xz*_ are represented by green and grey dashed lines, respectively. Inset: experimental set-up for transverse thermoelectric measurement. **c**, Comparison of the transverse thermopower and |*α*_*xz*_| with those of reported ANE-based materials and the mixed-dimensional conductor LaPt_2_B under zero magnetic field (*B* = 0 T)^11–13,16,17,36–38^. **d**, Temperature dependence of power factors and thermal conductivities (inset). **e**, Thermoelectric figure of merit *zT* as a function of temperature. **f**, Transverse thermoelectric figure of merit (*z*_*xz*_*T*) for our kagome metals compared to ANE-based systems, the artificially tilted multilayers Bi_88_Sb_12_/Bi_0.2_Sb_1.8_Te_3_, the goniopolar Re_4_Si_7_, and the mixed-dimensional conductor LaPt_2_B under zero magnetic field^11–13,16,17,25,38,41,42^. The data for ANE-based materials were estimated under zero-magnetic-field conditions, based on transverse thermopower measured in the remanent magnetization state. Source data

The goniopolar behaviour is experimentally examined by transport measurements. Evident anisotropy in the electrical resistivity is observed for LuCo_6_Ge_6_, which shows metallic behaviour with increasing temperature (Supplementary Fig. 10). Temperature-dependent Seebeck and Hall coefficients are depicted in Fig. 3a. The in-plane Seebeck coefficient *S*_IP_ remains consistently negative, hovering around −15 μV K^−1^ across the temperature range. Conversely, the out-of-plane Seebeck coefficient *S*_OP_ shows positive values that increase with temperature, ranging from 0 to 9.1 μV K^−1^ between 60 K and 300 K. The in-plane and out-of-plane Hall resistivities and coefficients show the same signs as the Seebeck coefficients (Supplementary Fig. 10 and inset of Fig. 3a), demonstrating electron transport in the in-plane direction and hole transport in the out-of-plane direction. We have also examined the transport properties of LuCr_6_Ge_6_ and HoCr_6_Ge_6_ kagome metals (Supplementary Figs. 11 and 12). They show the opposite goniopolar behaviour—hole conduction along the in-plane axis and electron conduction along the out-of-plane axis.

Goniopolarity can induce a transverse thermoelectric response under zero magnetic field^17^. Upon applying a temperature gradient along the longitudinal direction (inset of Fig. 3b), longitudinal *S*_L_ and goniopolar transverse *S*_*xz*_ thermopowers were generated in LuCo_6_Ge_6_ (Fig. 3b). The negligible transverse temperature difference suggests that *S*_*xz*_ primarily originates from goniopolarity, with negligible contributions from off-diagonal thermal conductivity and Seebeck effect to *S*_*xz*_ (Supplementary Fig. 13). *S*_*xz*_ exhibits a temperature-dependent increase, achieving 7 μV K^−1^ at 60 K and 18.4 μV K^−1^ at 300 K, surpassing the highest recorded room-temperature ANE thermopower in ANE-based materials^12^. The low *S*_L_ values remain consistent across the temperature range, and both the experimental *S*_L_ and *S*_*xz*_ align well with the calculated values (Fig. 3b). LuCr_6_Ge_6_ and HoCr_6_Ge_6_ also demonstrate exceptional *S*_*xz*_ of 7.3 μV K^−1^ and 8.1 μV K^−1^ at 300 K, respectively. Furthermore, a large transverse Peltier conductivity |*α*_*xz*_| of 105 A m^−1^ K^−1^ at 300 K was achieved, primarily due to the high electrical conductivity and large *S*_*xz*_. The parameter |*α*_*xz*_| is crucial, as it quantifies the induction of an electric current along the *x* axis in response to a temperature gradient applied along the *z* axis^35^. It is worth noting that both *S*_*xz*_ and |*α*_*xz*_| in LuCo_6_Ge_6_ are comparable to those reported in ANE-based systems under zero field^11–13,16,17,36–38^ (Fig. 3c). Figure 3d shows the temperature-dependent longitudinal and goniopolar transverse power factors. Across a broad temperature range, the goniopolar transverse power factor surpasses the longitudinal one, attaining 8.2 μW cm^−1^ K^−2^ at room temperature, comparable to some leading thermoelectric semiconductors^39,40^. LuCo_6_Ge_6_ shows notable anisotropy in total thermal conductivities, as evidenced by the inset in Fig. 3d, where the out-of-plane and in-plane thermal conductivities show values of 69 W m^−1^ K^−2^ and 11 W m^−1^ K^−2^ at 300 K, respectively. On the basis of the Wiedemann–Franz law, the lattice thermal conductivity was determined. Across the entire temperature range, the in-plane lattice thermal conductivity remains lower than that of the out-of-plane direction. The slight in-plane lattice distortion may suppress phonon propagation (Fig. 2f), thereby contributing to a low in-plane lattice thermal conductivity in LuCo_6_Ge_6_. Nevertheless, the in-plane lattice thermal conductivity remains higher than that of several thermoelectric semiconductors, which can be decreased by incorporating defect structures. Combining the high goniopolar transverse power factor and anisotropic thermal conductivities, we obtained a high goniopolar transverse thermoelectric figure of merit *z*_*xz*_*T* value of 1.15 × 10^−2^ at 300 K for LuCo_6_Ge_6_ (Fig. 3e), exceeding those of all previously reported ANE-based systems by an order of magnitude under zero field^11–13,16,17,25,38,41,42^ (Fig. 3f). Obviously, outstanding goniopolar kagome metals are expected to exhibit high electrical conductivities characterized by low carrier concentration and high mobility, along with large *S*_*xz*_ and low thermal conductivities (Supplementary Table 1). The reproducibility of the goniopolar behaviour and thermoelectric performance has been verified across different batches of samples (Supplementary Figs. 14 and 15). The differences in sample performance may be due to differences in carrier concentrations between crystals. Moreover, LuCr_6_Ge_6_ and HoCr_6_Ge_6_ also demonstrate exceptional transverse thermoelectric performance (Fig. 3f and Supplementary Figs. 16 and 17).

## Origin of the goniopolarity in LuCo_6_Ge_6_ and its siblings

**Fig. 4:**
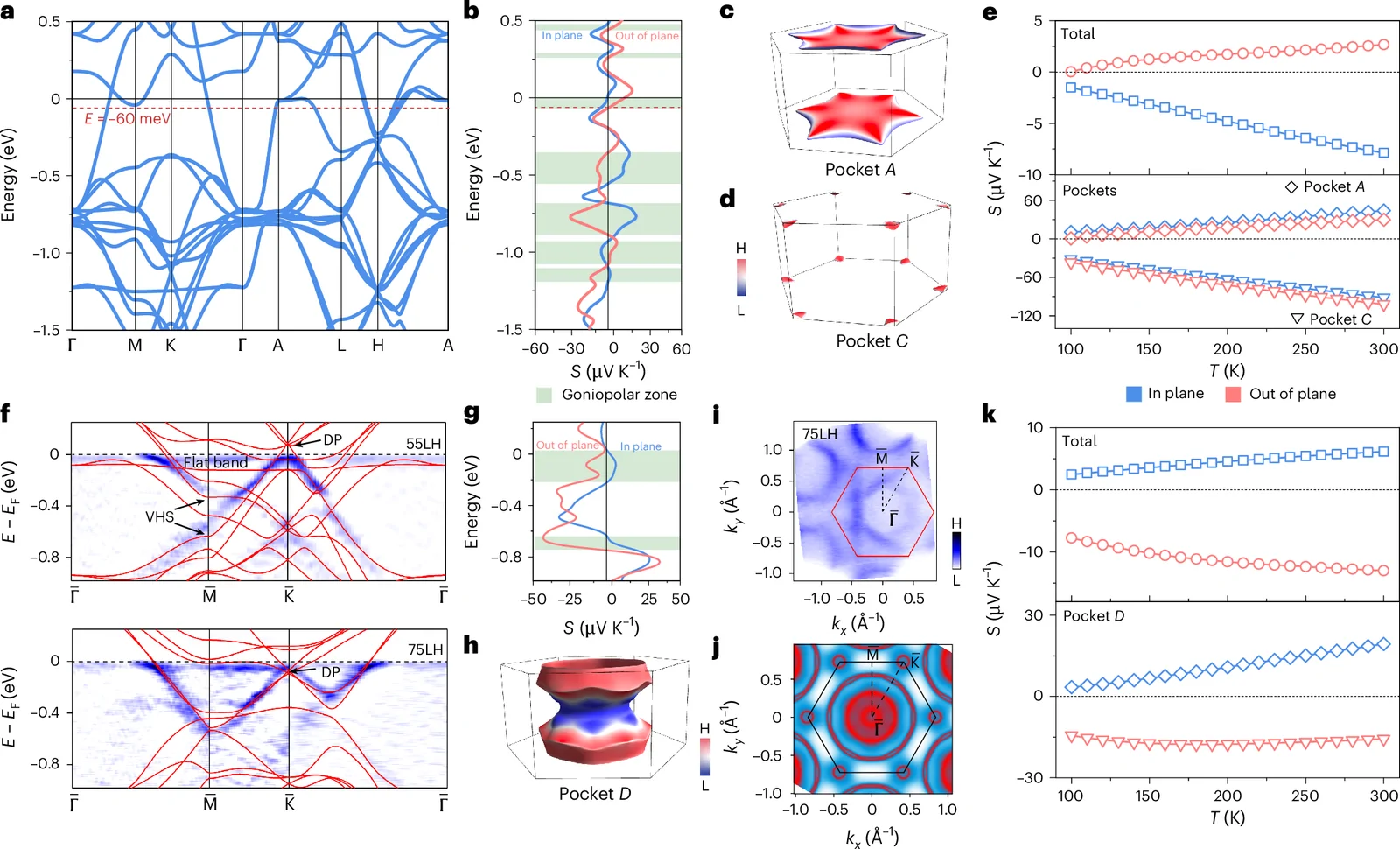
Theoretical analysis of goniopolarity. **a**, Band structure of LuCo_6_Ge_6_. **b**, The energy-dependent Seebeck coefficients for LuCo_6_Ge_6_. The red dashed lines represent the real Fermi level. **c**,**d**, The hole-dominated Fermi pocket A (**c**) and the electron-dominated pocket C (**d**) of LuCo_6_Ge_6_. **e**, Calculated Seebeck coefficients for all pockets (top) and each individual pocket (bottom) in LuCo_6_Ge_6_. **f**, Second derivatives of the ARPES spectra of LuCr_6_Ge_6_ (*T* = 15 K) along the $\bar{{\Gamma}}$−$\bar{\mathrm{K}}$−$\bar{\mathrm{M}}$−$\bar{\Gamma}$ lines measured by the 55-eV (top) and 75-eV (bottom) photons with linear horizontal (LH) polarization. The red solid curves are the DFT calculated bulk bands in *k*_*z*_ = 0 (top) and *π* (bottom) planes. As highlighted by the black arrows, multiple Dirac points and VHSs revealed in experiments are well reproduced by the calculations. **g**, The energy-dependent Seebeck coefficients for LuCr_6_Ge_6_. **h**, The hyperbolic pocket D of LuCr_6_Ge_6_. L and H denote low and high Fermi velocities, respectively. **i**, The constant-energy ARPES image of LuCr_6_Ge_6_ (*hν* = 75 eV, LH polarization) obtained by integrating the photoemission intensity within *E*_F_ = ± 20 meV. The red solid curves indicate the (001)-projected Brillouin zone. L and H denote low and high DOS, respectively. **j**, Calculated Fermi surface counter. **k**, Calculated Seebeck coefficients for all pockets (upper) and pocket D (lower) in LuCr_6_Ge_6_. Source data

To elucidate the origin of goniopolarity in LuCo_6_Ge_6_ and its sibling compounds Lu(Ho)Cr_6_Ge_6_, we investigated their band structures using density functional theory (DFT) calculations and angle-resolved photoemission spectroscopy (ARPES). As depicted in Fig. 4a and Supplementary Fig. 18, the band structure of LuCo_6_Ge_6_ shows hallmark features of kagome lattice such as Dirac points, VHSs, and flat bands near the Fermi level. The theoretical energy-dependent Seebeck coefficients demonstrate evident goniopolar behaviour in various energy zones due to the presence of VHSs and flat bands (Fig. 4b). On the basis of experimental and theoretical Seebeck coefficients, we conclude that the system is hole-doped, with the band dispersion shifted by about 60 meV below the Fermi level *E*_F_. This estimation was further corroborated by low-temperature specific heat analysis (Supplementary Fig. 4). The derived electronic specific-heat coefficient of *γ* = 0.01754 J mol^**−**1^ K^**−**2^ corresponds to an experimental DOS of *g*_exp_ = 7.44 (states per eV). DFT calculations within the proposed energy range indicate a DOS of ~7.05 states per eV, aligning well with the experimental approximation.

To further understand the goniopolar responses in LuCo_6_Ge_6_, we conducted theoretical calculations of Fermi pockets (labelled as A, B and C) at *E*_F_ = −60 meV and the corresponding axis-dependent Seebeck coefficients (Fig. 4c–e and Supplementary Fig. 18). The quasi-one-dimensional Fermi pocket A originating from the flat band near *E*_F_, localized at the *k*_*z*_ = π plane, shows hole-like dispersion, while pockets B and C exhibit electron-like characteristics. The calculated in-plane (*S*_cIP_) and out-of-plane (*S*_cOP_) Seebeck coefficients at *E*_F_ = −60 meV reveal a notable anisotropy: *S*_cOP_ shows positive values (hole-dominated transport), while *S*_cIP_ remains negative (electron-dominated behaviour), in agreement with experimental observations (Fig. 4e). The hole-dominated pocket A consistently shows positive values along all crystallographic axes, while the electron-dominated pockets B and C consistently show negative values. The total Seebeck coefficient (*S*_total_) is calculated as a conductivity-weighted average across all Fermi pockets: ${S}_{\mathrm{total}}=\frac{{\sum }_{i=1}^{n}{S}_{i}{\sigma }_{i}}{{\sum }_{i=1}^{n}{\sigma }_{i}}$, where *S*_*i*_ and *σ*_*i*_ represent the Seebeck coefficient and electrical conductivity of the *i*th pocket, respectively^43^. Analysis reveals that the contribution of pocket B is negligible due to its low *S*_*i*_ value. In the in-plane direction, pockets A and C exhibit similar conductivity (Supplementary Fig. 18), yet the negative *S*_C_ outweighs the positive *S*_A_, yielding a negative *S*_IP_. In the out-of-plane direction, the conductivity of pocket A (*σ*_A_) surpasses that of pocket C by a factor of ~6, amplifying the positive *S*_A_ contribution and resulting in a positive *S*_OP_. This synergy between the flat-band-related pocket A and the isotropic pocket C highlights a flat-band-driven two-band goniopolar mechanism in LuCo_6_Ge_6_.

In contrast to the LuCo_6_Ge_6_ metal, the band structures of LuCr_6_Ge_6_ and HoCr_6_Ge_6_ are similar, which were investigated using synchrotron-based ARPES and DFT calculations (Supplementary Figs. 19 and 20). Figure 4f shows the second derivatives of the ARPES spectra of LuCr_6_Ge_6_ (*T* = 15 K) measured along the $\bar{\Gamma}$ − $\bar{\mathrm{K}}$ − $\bar{\mathrm{M}}$ − $\bar{\Gamma}$ high-symmetry path by the 55-eV (Fig. 4f, top) and 75-eV (Fig. 4f, bottom) photons with linear horizontal polarization. The theoretical band structure shows typical flat bands and VHSs near the Fermi level, leading to the Seebeck coefficients displaying goniopolar behaviour in various energy zones (Fig. 4g and Supplementary Fig. 19). The goniopolar response of LuCr_6_Ge_6_ also shows strong *E*_F_ dependence, similar to the tunability observed in LuCo_6_Ge_6_. By correlating the energy of the Dirac point in *k*_*z*_ = π plane with the experimental spectra, the real Fermi level was determined at *E*_F_ = −0.1 eV (Fig. 4f and Supplementary Fig. 19). As indicated by the appended Brillouin zone and labelled high-symmetry points ($\bar{\Gamma}$, $\bar{\mathrm{K}}$ and $\bar{\mathrm{M}}$), the measured Fermi surface exhibits a characteristic hexagonal topology, in agreement with theoretical calculations (Fig. 4i,j). The DFT calculations reveal four anisotropic Fermi pockets (labelled as D, E, F and G) at *E*_F_ = −0.1 eV (Fig. 4h and Supplementary Fig. 19), where the hyperbolic pocket D and bowl-like pocket E are more than half-filling, while open pockets F and G are less than half-filling, attributed to the 3*d* orbitals of the Cr-based kagome lattice. At *E*_F_ = −0.1 eV, LuCr_6_Ge_6_ displays pronounced anisotropy in the calculated Seebeck coefficients, showing positive in-plane values but negative out-of-plane values, in contrast to LuCo_6_Ge_6_ (Fig. 4k). The axis-dependent Seebeck coefficients were calculated for four Fermi pockets, as depicted in Fig. 4k and Supplementary Fig. 19. The hyperbolic pocket D and bowl-like pocket E yield positive values along the in-plane direction but negative values along the out-of-plane direction. Conversely, open pockets F and G show uniform polarity across all crystallographic axes. The negligible contribution of pockets F and G is attributed to their low *S*_*i*_ values based on the conductivity-weighted equation. Either pocket D or E alone is sufficient to produce the observed goniopolar behaviour, distinct from the flat-band-driven two-band mechanism, and instead points to a VHS-enabled single-band mechanism in LuCr_6_Ge_6_. A detailed analysis of the Fermi surface evolution shows that no abrupt topological transition or pocket reconnection occurs exactly at the onset or disappearance of goniopolarity (Supplementary Fig. 20). The electronic structure and goniopolar behaviour of HoCr_6_Ge_6_ closely resemble those of LuCr_6_Ge_6_, further emphasizing a single-band mechanism (Supplementary Fig. 21). Hence, we can achieve both single-band and two-band mechanisms in kagome systems. These findings underscore the pivotal role of fermiology in tailoring emergent transport phenomena in kagome metals.

## Outlook

To summarize, we demonstrate that the electronic band structures of kagome metals inherently support goniopolarity through two distinct mechanisms: a flat-band-enabled two-band mechanism realized in LuCo_6_Ge_6_ and a VHS-driven single-band mechanism observed in Lu(Ho)Cr_6_Ge_6_. Goniopolar thermoelectricity highlights the versatility of kagome lattices in tailoring transport phenomena, beneficial to not only energy conversion but also the emergent topological quantum states. The absence of magnetic fields eliminates stray-field interference, positioning the goniopolar thermoelectric technology as a promising route for waste heat recovery and solid-state cooling. Furthermore, the fermiology-driven framework can, in principle, be extended to other kagome materials and potentially to a broader class of topological quantum materials. This work establishes a conceptual bridge between kagome fermiology and goniopolarity, expanding the role of kagome materials from hosting exotic quantum states to enabling emergent transport design.

## Methods

### Sample synthesis

Single crystals of LuCo_6_Ge_6_ were synthesized using the Sn-flux growth method. A mixture of Lu_1.5_Co_6_Ge_18_Sn_100_ high-purity elements was placed in an alumina crucible, sealed in a quartz ampoule under partial argon pressure, and heated to 1,373 K over 10 h. The temperature was maintained for 48 h before gradually cooling to 973 K at a rate of 2 K h^−1^. The single crystals were then separated from the flux using a centrifugal process, resulting in bar-shaped crystals with shiny surfaces. The preparation of LuCr_6_Ge_6_ and HoCr_6_Ge_6_ single crystals followed the same method.

### Sample characterization

Laue X-ray diffraction was conducted to confirm the single-crystalline nature of the as-grown crystals at room temperature. STEM specimens of LuCo_6_Ge_6_, LuCr_6_Ge_6_ and HoCr_6_Ge_6_ were prepared using a Tescan dual beam SEM focused ion beam (SEM-FIB). After FIB thinning, the specimens underwent further low-energy Ar^+^ ion milling using a Fischione 1040 Nanomill. Final surface cleaning and thinning start from 700 eV and gradually decreases to 300 eV. HAADF-STEM and EDS were performed using a JEOL JEM-ARM300F transmission electron microscope at 300 kV. The microscope was equipped with double aberration correctors, Gatan Image Filter Quantum with Gatan K2 Summit and dual 100 mm^2^ Si drift detectors. The probe convergence semi-angle for HAADF-STEM images is 25.7 mrad, with a large inner collection angle of 70 mrad. For EDS measurements, to avoid overlapping between different elements, L-line was used for Lu, with K-lines for Cr, Co and Ge. To measure the ellipticity of the atomic column, we first fit the atomic column with a 2D Gaussian function with Atomap^44^. The standard deviation along two orthogonal directions, *c*_*x*_ and *c*_*y*_, is then collected, whose ratio determines the ellipticity and elongated direction. Specifically, when *c*_*x*_ > *c*_*y*_, the ellipticity was calculated as *c*_*x*_/*c*_*y*_; when *c*_*x*_ < *c*_*y*_, the ellipticity was calculated as *c*_*y*_/*c*_*x*_.

### ARPES measurements

High-resolution ARPES measurements of LuCr_6_Ge_6_ and HoCr_6_Ge_6_ were performed at the APE-LE beamline in Elettra Sincrotrone Trieste and the 1^2^-ARPES end station of UE-112-PGM2 beamline at Helmholtz Zentrum Berlin BESSY-II light source. The energy and angular resolutions were set to better than 10 meV and 0.1°, respectively. Samples were cleaved in situ, yielding flat mirror-like (001) surfaces. During the experiments, the temperature was kept at 15–26 K if not specified otherwise, and the vacuum conditions were maintained better than 7.0 × 10^−11^ torr. We used linear horizontal polarization for all the measurements.

### Transport property measurements

The longitudinal and Hall resistivities of kagome metals were measured using a physical property measurement system (Quantum Design) equipped with the electrical transport option. The Hall carrier concentration (*n*) was determined from the slope of the magnetic-field dependence of the Hall resistivity. Hall mobility (*μ*) values were calculated from the equation 1/*ρ* = *eμn* (where *e* represents the elementary charge). The in-plane (*S*_IP_) and out-of-plane (*S*_OP_) Seebeck coefficients, as well as the in-plane (*κ*_IP_) and out-of-plane (*κ*_OP_) thermal conductivities of kagome metals, were measured using a standard one-heater, two-thermometer steady-state method in a physical properties measurement system under high vacuum conditions. To create a temperature gradient, the sample was affixed to a heat sink with a heater. Chromel–constantan thermocouples were used to measure temperature and voltage differences. Raw data were field-symmetrized for resistivity, Seebeck coefficient and thermal conductivity, and antisymmetrized for Hall resistivity to account for contact misalignment. In-plane and out-of-plane transport properties were measured by applying a current or temperature gradient along the in-plane (001) and out-of-plane $\left({\overline{1}}00\right)$ directions, respectively.

After obtaining *S*_IP_ and *S*_OP_, transverse thermopower measurements were conducted using chromel–constantan thermocouples to measure longitudinal thermopowers *S*_L_, longitudinal temperature difference, and transverse temperature difference (shown in the inset of Fig. 3b). Copper wires were used to measure transverse thermopowers *S*_*xz*_. The sign of the transverse voltage is determined by the electrode definition (*V*+ and *V−*) and the measurement geometry (inset of Fig. 3b). Thus, the absolute sign of *S*_*xz*_ holds no physical significance. The temperature gradient is homogeneously oriented at a 45° angle from the in-plane (*x*) and out-of-plane (*z*) axes. The experimental transverse thermopowers are calculated as follows: *S*_*xz*_ = *E*_T_/∇*T*_L_ = (∆*V*_T_/*W*)/(∆*T*_L_/*L*), where ∆*V*_T_, *W*, ∆*T*_L_ and *L* represent the transverse voltage difference, transverse width, longitudinal temperature difference and longitudinal length, respectively. The theoretical *S*_*xz*_ and *S*_L_ (dashed lines) in Fig. 3b are determined through the equations *S*_*xz*_ = (*S*_OP_ − *S*_IP_)/2, and *S*_L_ = (*S*_OP_ + *S*_IP_)/2, respectively. We evaluated the transverse Peltier conductivity |*α*_*xz*_| using the experimental *S*_L_ and *S*_*xz*_ values for kagome metals as follows: $|{\alpha }_{xz}|=\frac{1}{2}({\sigma }_{{\rm{IP}}}+{\sigma }_{{\rm{OP}}}){S}_{xz}+\frac{1}{2}({\sigma }_{{\rm{IP}}}-{\sigma }_{{\rm{OP}}}){S}_{{\rm{L}}}$, where *σ*_IP_ and *σ*_OP_ represent in-plane and out-of-plane electrical conductivities, respectively. The in-plane power factor PF_IP_, out-of-plane power factor PF_OP_ and transverse power factor PF_*xz*_ can be calculated as PF_IP_ = *σ*_IP_*S*_IP_^2^, PF_OP_ = *σ*_OP_*S*_OP_^2^ and ${{\rm{PF}}}_{yx}=\frac{4{S}_{xz}^{2}}{{\left(\sqrt{{\rho }_{\mathrm{IP}}}+\sqrt{{\rho }_{\mathrm{OP}}}\right)}^{2}}$, respectively. Finally, the in-plane *z*_IP_*T*, out-of-plane *z*_OP_*T* and transverse *z*_*xz*_*T* figure of merits can be obtained as: *z*_IP_*T* = PF_IP_*T*/*κ*_IP_, *z*_OP_*T* = PF_OP_*T*/*κ*_OP_ and ${z}_{xz}T=\frac{4{S}_{xz}^{2}T}{{\left(\sqrt{{\rho }_{\mathrm{IP}}{\kappa }_{\mathrm{IP}}}+\sqrt{{\rho }_{\mathrm{OP}}{\kappa }_{\mathrm{OP}}}\right)}^{2}}$, respectively. The low-temperature (2–300 K) heat capacity *C*_*p*_ was measured using a PPMS DynaCool (Quantum Design) instrument.

Magnetization measurements were conducted on our samples using a magnetic property measurement system (MPMS3, Quantum Design) across the temperature range of 2–300 K (Supplementary Figs. 4–6). LuCo_6_Ge_6_ and LuCr_6_Ge_6_ exhibited consistent paramagnetic behaviour without any evidence of magnetic ordering or fluctuations. HoCr_6_Ge_6_ showed a magnetic transition at ~4 K, showing paramagnetic characteristics above this temperature^45^. Thus, all three compounds are unequivocally in the paramagnetic state within the goniopolar temperature ranges.

### Theoretical calculations

First-principles calculations are carried out based on DFT^46,47^ as implemented in the Vienna ab initio simulation package (VASP)^48,49^ with the projector augmented wave method^50^. The generalized gradient approximation (GGA) within Perdew–Burke–Ernzerbof scheme is adopted to describe the exchange–correlation energy functional^51^. The cut-off energy is set to be 500 eV. A Γ-centred *k*-mesh of 12 × 12 × 5 is adopted to sample the Brillouin zone. The energy and force convergence criteria are set to be 10^−6^ eV and 10^−3^ eV Å^−1^, respectively. With the obtained DFT results, the maximally localized Wannier functions are constructed using WANNIER90 package^52^, including the *d* orbitals of Lu/Ho atoms and kagome Cr/Co atoms, and *p* orbitals of Ge atoms. The thermoelectric properties are analysed based on Boltzmann module^53^ and self-made codes. A constant relaxation time of 80 fs and a *k*-mesh of 200 × 200 × 200 is adopted to ensure the convergence. The Fermi surfaces are processed using WannierTools package^54^ and visualized via Fermisurfer^55^. Without specific notation, the presented results are shown without spin–orbit coupling.

## Online content

Any methods, additional references, Nature Portfolio reporting summaries, source data, extended data, supplementary information, acknowledgements, peer review information; details of author contributions and competing interests; and statements of data and code availability are available at https://doi.org/10.1038/s41563-026-02678-4.

## Supplementary information

Supplementary Information Supplementary Note, Figs. 1–21 and Table 1.

Supplementary Data 1 Atomic coordinates of the computational models.

## Source data

Source Data Fig. 1 Source data for Fig. 1a,b,e,h.

Source Data Fig. 3 Source data for Fig. 3a–f

Source Data Fig. 4 Source data for Fig. 4a,b,e–g,k.

## Data Availability

All relevant data supporting the findings of this study are available within the article and its Supplementary Information. Source data are provided with this paper.
