# Supplementary material for: Fermiology-driven goniopolar transverse thermoelectricity in kagome metals
Source: Nat Mater. 2026 Jul 31;25(10):1783–90. doi: 10.1038/s41563-026-02678-4 (PMC13619446; doi:10.1038/s41563-026-02678-4)
Supplement: Supplementary file 1 [file 41563_2026_2678_MOESM1_ESM.pdf]

nature materials

Supplementary information https://doi.org/10.1038/s41563-026-02678-4

# Fermiology-driven goniopolar transverse thermoelectricity in kagome metals

In the format provided by the authors and unedited

# Table of Contents

**This Supplementary Information file archives:**

1. Supplementary note describes the thermoelectric response, single s-orbital kagome model, and transport of flat band and hyperbolic band.

2. Supplementary Figs. 1-21 are chronologically cited in the main text.

**Supplementary Figs. 1-3** and **Supplementary Figs. 18-21** present the theoretical calculation results.

**Supplementary Figs. 4-17** show the additional experiment data.

3. Supplementary Table 1 shows the transport properties of all samples.

**Supplementary Note**

## Thermoelectric response

The current density $\boldsymbol{J}$ and the heat current $\boldsymbol{J_Q}$ in the presence of an electric field $\boldsymbol{E}$ and a temperature gradient $\nabla T$ are given by

$$\boldsymbol{J} = \boldsymbol{\sigma}(\boldsymbol{E} - \boldsymbol{S}\boldsymbol{\nabla}T), \tag{1}$$

$$\boldsymbol{J_Q} = T\boldsymbol{\alpha}\boldsymbol{E} - \boldsymbol{K}\boldsymbol{\nabla}T, \tag{2}$$

where the electrical conductivity $\boldsymbol{\sigma}$, the Peltier conductivity $\boldsymbol{\alpha}$, the Seebeck coefficient $\boldsymbol{S}$ and the thermal conductivity $\boldsymbol{K}$ are 3 × 3 tensors. The Peltier conductivity is related to the thermoelectric tensor by $\boldsymbol{S} = \boldsymbol{\sigma}^{-1} \cdot \boldsymbol{\alpha}$.

According to the Boltzmann theory, the transport tensors can be obtained from the distributions,

$$\sigma(T;E) = \int \sigma(\varepsilon) f(\varepsilon, T) d\varepsilon, \tag{3}$$

$$\alpha(T;E) = \frac{1}{eT}\int (\varepsilon - E)\sigma(\varepsilon) f(\varepsilon, T) d\varepsilon. \tag{4}$$

where $\sigma(\varepsilon) = e^2\tau(\varepsilon)v^2(\varepsilon)g(\varepsilon)$ and $g(\varepsilon)$ is the density of states. With the Sommerfeld expansion, the Peltier conductivity can be given as

$$\alpha = \frac{\pi^2}{3}\frac{k_b^2 T}{e}\frac{d\sigma}{d\varepsilon}|_{\varepsilon=E}. \tag{5}$$

Therefore, the Seebeck response is given by

$$S = \sigma^{-1}\alpha = \frac{\pi^2}{3}\frac{k_b^2 T}{e}\frac{d\ln\sigma}{d\varepsilon}|_{\varepsilon=E} \tag{6}$$

$$= \frac{\pi^2}{3}\frac{k_b^2 T}{e}\Big(\frac{d\ln g}{d\varepsilon} + \frac{d\ln v^2}{d\varepsilon} + \frac{d\ln\tau}{d\varepsilon}\Big)|_{\varepsilon=E} \tag{7}$$

$$= \frac{\pi^2}{3}\frac{k_b^2 T}{e}\Big(\frac{g'}{g} + (\frac{1}{2}mv^2)^{-1} + \frac{\tau'}{\tau}\Big)|_{\varepsilon=E}. \tag{8}$$

The first term represents the contribution from the density of states (DOS) distribution—implying that a rapid change in DOS leads to a large Seebeck response. Typical examples include band edges in semiconductors as well as regions with high DOS, such as flat bands and VHSs. The second term reflects the kinetic susceptibility, indicating how sensitive the system is to changes in kinetic energy. Particles with lower kinetic energy exhibit a greater sensitivity to response. The third term accounts for the effect of an asymmetric distribution of the relaxation time. A longer lifetime for higher-energy states contributes to a positive Seebeck response, while a shorter lifetime leads to a negative response. In this study, the relaxation time $\tau$ is treated as a constant under the relaxation time approximation. Therefore, the Seebeck response is primarily determined by the DOS distribution and the kinetic energy.

## Single s-orbital kagome model

Kagome systems exhibit fascinating properties because of the interesting electronic states at varying fillings, such as flat bands, van hove singularities, and Dirac points. For single s-orbital kagome lattice, with the inclusion of nearest in-plane and out-of-plane hoppings, the Hamiltonian is given by

$$H = \begin{pmatrix} t_o\cos k_z c & 2t_i\cos\left[(\frac{k_x}{2}+\frac{\sqrt{3}k_y}{2})a\right] & 2t_i\cos\left[(\frac{k_x}{2}-\frac{\sqrt{3}k_y}{2})a\right] \\ 2t_i\cos\left[(\frac{k_x}{2}+\frac{\sqrt{3}k_y}{2})a\right] & t_o\cos k_z c & 2t_i\cos k_x a \\ 2t_i\cos\left[(\frac{k_x}{2}-\frac{\sqrt{3}k_y}{2})a\right] & 2t_i\cos k_x a & t_o\cos k_z c \end{pmatrix} \tag{9}$$

where $t_{i(o)}$ stands for the nearest IP (OP) hopping. The eigenvalues are

$$E_0 = \left[t_o\cos k_z - 2t_i,\ t_o\cos k_z + t_i\big(1 \pm \sqrt{3 + 2\cos 2k_x + 4\cos k_x\cos\sqrt{3}k_y}\big)\right]. \tag{10}$$

With the inclusion of transport in the third direction, the transport behavior is significantly modified. In particular, the divergence is suppressed, resulting in less pronounced electron correlations.

## Transport of flat bands

With only nearest hoppings, one can obtain perfect flat bands $E_{\mathrm{FB}} = t_o \cos k_z - 2t_i$ without transport in the plane. Flat bands always exhibit interesting properties due to a significant increase of DOS, which contributes to the enhancement of electron correlation and Fermi surface instability. For 2D flat bands in 3D space, the DOS is given by

$$g_{\mathrm{FB}} = \int \delta(E-\varepsilon)d\boldsymbol{k} = \frac{2}{\pi c\sqrt{t_o^2 - E^2}}, \tag{11}$$

where $E = E_0 + 2t_i$. The derivative of DOS is

$$g_{\mathrm{f}}'(E) = \quad \frac{2E}{\pi c(t_o^2 - E^2)^{\frac{3}{2}}} \tag{12}$$

The DOS and its derivative denote the divergence at $E = \pm t_0$ in proximity to empty or full occupation, as shown in fig. S2. It only conducts along the normal direction with the conductivity given by

$$\sigma_{\mathrm{FBz}} = e^2 \int \tau(\boldsymbol{k})v_z(\boldsymbol{k})v_z(\boldsymbol{k})\Big[-\frac{\partial f}{\partial \varepsilon}\Big]d\boldsymbol{k} = \frac{\tau e^2 c}{\pi}\sqrt{t_o^2 - E^2} \tag{13}$$

within the constant relaxation-time approximation. The group velocity is

$$v_z = -t_o \sin k_z \tag{14}$$

and the out-of-plane mass is

$$m_z^{-1} = \frac{dv}{dk} = -t_o \cos k_z. \tag{15}$$

In this manuscript, $\hbar$ is set to 1. Using the Sommerfeld expansion, the thermoelectric coefficient can be given by

$$\alpha \approx \frac{\pi^2}{3}\frac{k_b^2 T}{e}\frac{d\sigma_{\mathrm{FBz}}}{d\varepsilon}|_{\varepsilon=E} = \frac{\pi^2}{3}\frac{k_b^2 T}{e}\frac{\tau e^2 c}{\pi}\frac{-E}{\sqrt{t_o^2 - E^2}}. \tag{16}$$

Therefore, the Seebeck coefficient is

$$\begin{aligned} S_z =& \frac{\pi^2}{3}\frac{k_b^2 T}{e}\frac{d\ln\sigma}{d\varepsilon}|_{\varepsilon=E} \\ =& \frac{\pi^2}{3}\frac{k_b^2 T}{e}\Big(\frac{g'}{g} + (\frac{1}{2}mv^2)^{-1}\Big) \\ =& \frac{\pi^2}{3}\frac{k_b^2 T}{e}\Big(\frac{E}{t_o^2 - E^2} + \frac{-2E}{t_o^2 - E^2}\Big) \\ =& \frac{\pi^2}{3}\frac{k_b^2 T}{e}\frac{-E}{t_o^2 - E^2}, \end{aligned} \tag{17}$$

indicating the large Seebeck response in proximity to empty or full occupations as shown in fig. S2.

## Transport of hyperbolic band

To capture goniopolar transport close to VHS, the Hamiltonian for a hyperbolic band is taken as

$$H_0 = c_1 k_x^2 + c_2 k_y^2 - c_3 k_z^2. \tag{18}$$

where $c_i$ are positive. The density of state is given by

$$g = \int \delta(E - H_0)d\boldsymbol{k} \tag{19}$$

Taking the polar coordinate transformation $q_x = \frac{\rho}{\sqrt{c_1}}\cos\theta$ and $q_y = \frac{\rho}{\sqrt{c_2}}\sin\theta$, we then have $dk_x dk_y = \frac{1}{\sqrt{c_1 c_2}}\rho d\rho d\theta$,

$$\begin{aligned} g =& \frac{2\pi}{V\sqrt{c_1c_2}}\int_\Omega \delta(E-\rho^2+c_3k_z^2)\rho d\rho dk_z \\ =& \frac{2\pi}{V\sqrt{c_1c_2}}\int_\Omega \frac{1}{2}\Theta(E+c_3k_z^2)\Theta(R^2-E-c_3k_z^2)dk_z, \end{aligned} \tag{20}$$

where Θ is the Heaviside step function and *V* is the volume of *k*-space. Taking the boundary for $k_z$ as $[-l_z, l_z]$, the DOS is given by

$$g = \frac{2\pi}{V\sqrt{c_1c_2}}\begin{cases} \frac{N}{2}(q_z-b_z), & -c_3q_z^2 \le E < 0, \\ \frac{N}{2}q_z, & 0 \le E \le R^2, \\ 0, & E < -c_3q_z^2 || E > R^2. \end{cases} \tag{21}$$

Here, N = 2 due to the presence of mirror symmetry $m_z$, and $b_z = \sqrt{\frac{-E}{c_3}}$ and $q_z = \mathrm{Min}[\sqrt{\frac{R^2-E}{c_3}}, l_z]$ since the Fermi surface is only defined on the surface $E-\rho^2+c_3k_z^2 = 0$ for $\rho \le R$. Apparently, the DOS can be directly obtained from the boundary of $k_z$.

Consequently, the derivative of DOS is then given by

$$g' = \frac{2\pi}{V\sqrt{c_1c_2}}\begin{cases} \frac{N}{2}(q_z'-b_z'), & -c_3q_z^2 \le E < 0, \\ \frac{N}{2}q_z', & 0 \le E \le R^2, \\ 0, & E < -c_3q_z^2 || E > R^2. \end{cases} \tag{22}$$

Here, $b_z' = -\frac{1}{2\sqrt{-Ec_3}}$, and $q_z' = [-\frac{1}{2\sqrt{c_3(R^2-E)}}, 0]$ for $[\sqrt{\frac{R^2-E}{c_3}}, l_z]$, separately. Obviously, $g'$ is positive for $E < 0$ and negative for $E > 0$, indicating a sudden sign change when crossing the VHS.

With the group velocity $v_i = s_i 2c_i k_i$ ($s_i = \{+, +, -\}$ for $i = x, y, z$), the IP conductivity is then given by

$$\begin{aligned} \sigma_{xx(yy)} =& \frac{1}{V\sqrt{c_1c_2}}\int c_{1(2)}^2\rho^3 d\rho\int_0^{2\pi} f(\theta)^2 d\theta \int \delta(E-\rho^2+c_3k_z^2)dk_z \\ =& \frac{\pi c_{1(2)}^2}{V\sqrt{c_1c_2}}\frac{1}{2}\int (E+c_3k_z^2)dk_z \\ =& \frac{\pi c_{1(2)}^2}{2V\sqrt{c_1c_2}}(Ek_z+\frac{c_3}{3}k_z^3)|_\Omega \end{aligned} \tag{23}$$

$$= \frac{\pi c_{1(2)}^2}{2V\sqrt{c_1c_2}}\begin{cases} N(Eq_z+\frac{c_3}{3}q_z^3-Eb_z-\frac{c_3}{3}b_z^3), & -c_3q_z^2 \le E < 0, \\ N(Eq_z+\frac{c_3}{3}q_z^3), & 0 \le E \le R^2, \\ 0, & E < -c_3q_z^2 || E > R^2, \end{cases} \tag{24}$$

where *f*(*θ*) is cos(*x*) for *x* direction and sin *θ* for *y* direction.

The OP conductivity is given by

$$\begin{aligned} \sigma_{zz} =& \frac{1}{V\sqrt{c_1c_2}}\int \rho d\rho \int_0^{2\pi}\sin^2\theta d\theta \int c_3^2k_z^2\delta(E-\rho^2+c_3k_z^2)dk_z \\ =& \frac{2\pi c_3^2}{V\sqrt{c_1c_2}}\frac{1}{2}\int k_z^2\Theta(E+c_3k_z^2)dk_z \\ =& \frac{2\pi c_3^2}{V\sqrt{c_1c_2}}\frac{1}{2}\frac{k_z^3}{3}\Theta(E+c_3k_z^2)|_\Omega \\ =& \frac{\pi c_3^2}{3V\sqrt{c_1c_2}}\begin{cases} N\left(q_z^3-b_z^3\right), & -c_3q_z^2 \le E < 0 \\ Nq_z^3 & 0 \le E \le R^2 \\ 0, & E < -c_3q_z^2 || E > R^2 \end{cases} \end{aligned} \tag{25}$$

Thus, the Peltier conductivity is

$$\begin{aligned}\alpha_{xx(yy)} =&\frac{\pi^2}{3}\frac{k_B^2 T}{e}\frac{d\sigma_{xx(yy)}}{d\varepsilon}|_{\varepsilon=E}\\ =&\frac{\pi^2}{3}\frac{k_B^2 T}{e}\frac{\pi c_{1(2)}^2}{2V\sqrt{c_1 c_2}}\begin{cases}N(Eq_z' + q_z + c_3 q_z^2 q_z' - Eb_z' - b_z - c_3 b_z^2 b_z'), & -c_3 q_z^2 \leq E < 0,\\ N(Eq_z' + q_z + c_3 q_z^2 q_z'), & 0 \leq E \leq R^2,\\ 0, & E < -c_3 q_z^2 || E > R^2,\end{cases}\end{aligned} \tag{26}$$

$$\begin{aligned}\alpha_{zz} =&\frac{\pi^2}{3}\frac{k_B^2 T}{e}\frac{d\sigma_{zz}}{d\varepsilon}|_{\varepsilon=E}\\ =&\frac{\pi^2}{3}\frac{k_B^2 T}{e}\frac{\pi c_3^2}{3V\sqrt{c_1 c_2}}\begin{cases}3N\Big(q_z^2 q_z' - b_z^2 b_z'\Big), & -c_3 q_z^2 \leq E < 0,\\ 3N q_z^2 q_z' & 0 \leq E \leq R^2,\\ 0, & E < -c_3 q_z^2 || E > R^2.\end{cases}\end{aligned} \tag{27}$$

The Seebeck response is then given by

$$S_{xx(yy)} =\frac{\pi^2}{3}\frac{k_B^2 T}{e}\begin{cases}\frac{Eq_z' + q_z + c_3 q_z^2 q_z' - Eb_z' - b_z - c_3 b_z^2 b_z'}{Eq_z + \frac{c_3}{3} q_z^3 - Eb_z - \frac{c_3}{3} b_z^3}, & -c_3 q_z^2 \leq E < 0,\\ \frac{Eq_z' + q_z + c_3 q_z^2 q_z'}{Eq_z + \frac{c_3}{3} q_z^3}, & 0 \leq E \leq R^2,\\ 0, & E < -c_3 q_z^2 || E > R^2,\end{cases} \tag{28}$$

$$S_{zz} =\frac{\pi^2}{3}\frac{k_B^2 T}{e}\begin{cases}\frac{3q_z^2 q_z' - 3b_z^2 b_z'}{q_z^3 - b_z^3}, & -c_3 q_z^2 \leq E < 0,\\ \frac{3q_z'}{q_z}, & 0 \leq E \leq R^2,\\ 0, & E < -c_3 q_z^2 || E > R^2.\end{cases} \tag{29}$$

According to Eq. 6, the Seebeck response can be ascribed to the contributions from the change of DOS and kinetic energy. For both IP and OP responses, the contributions from DOS change turns from positive to negative when crossing the VHS or boundaries. However, the kinetic contribution is still positive and increasing for in-plane transport, whereas it turns to negative for out-of-plane transport when the VHS or boundaries are crossed due to the depletion of high-kinetic-energy states.

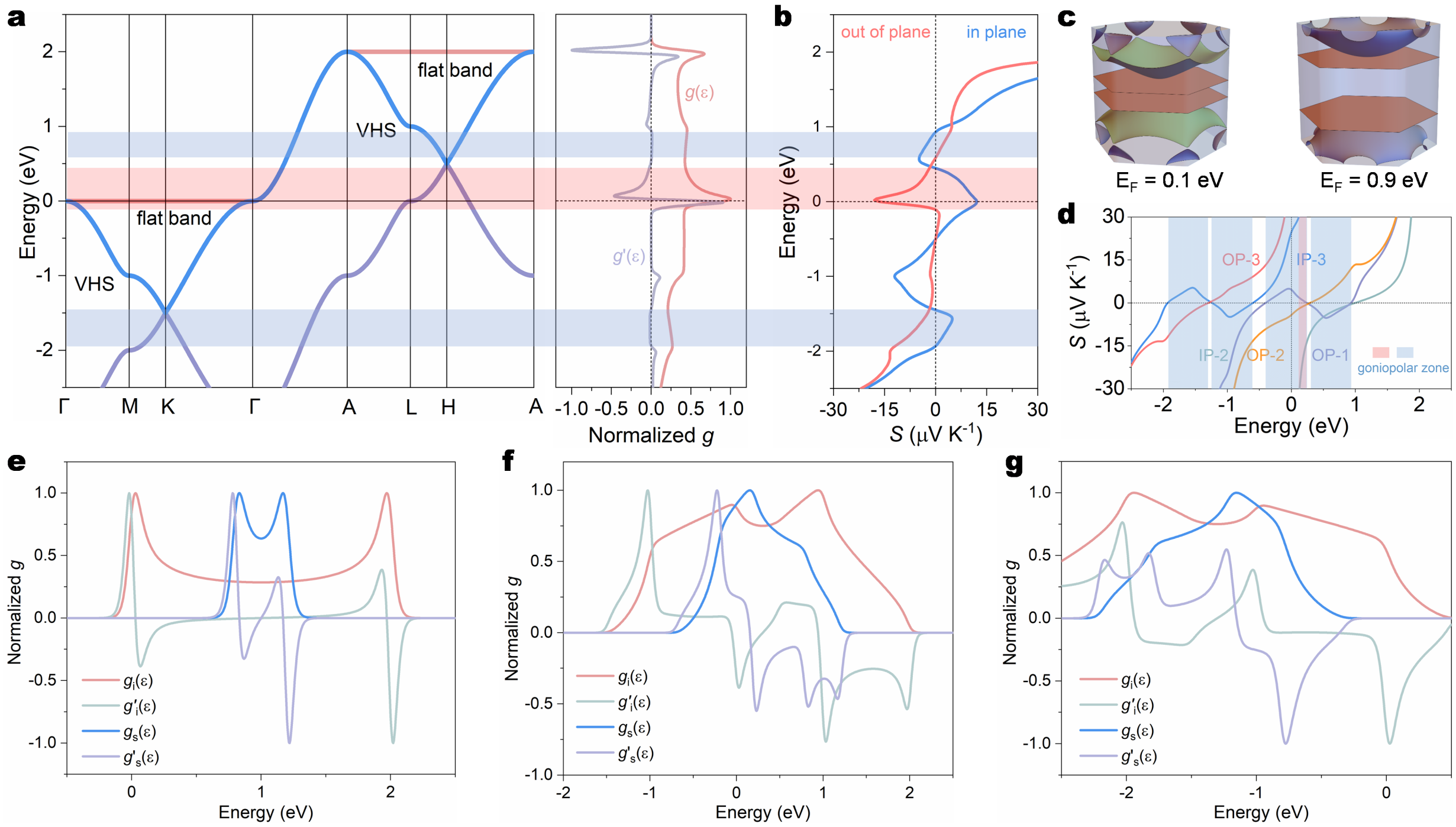


**Supplementary Fig. 1. Goniopolarity in kagome lattice. a**, Band structure of single-orbital kagome models featuring exclusively in-plane and out-of-plane nearest hoppings. The density of states $g(\varepsilon)$ and its derivative $g'(\varepsilon)$ are shown on the right side. The parameters are set as $a = 1$, $c = 1$, $t_i = -0.5$, $t_o = -1$. An intermediate interlayer hopping is implemented, which demonstrates that the goniopolar transport associated with kagome features remains robust. **b**, Corresponding in-plane $S_{IP}$ (blue) and out-of-plane $S_{OP}$ (red) Seebeck coefficients. The single-band and two-band goniopolar zones, marked in blue and red shading, exhibit opposite signs for in-plane and out-of-plane Seebeck response. These zones are positioned near flat bands and van Hove singularities (VHSs). **c**, Typical Fermi surface at $E_F$ = 0.1 eV and $E_F$ = 0.9 eV. **d**, Band-resolved Seebeck coefficients with shaded areas for single-band (blue) and two-band (red) goniopolar zones. **e-g**, Normalized density of states $g(\varepsilon)$ and their derivative $g'(\varepsilon)$ of red bands (**e**), blue bands (**f**), and purple bands (**g**).

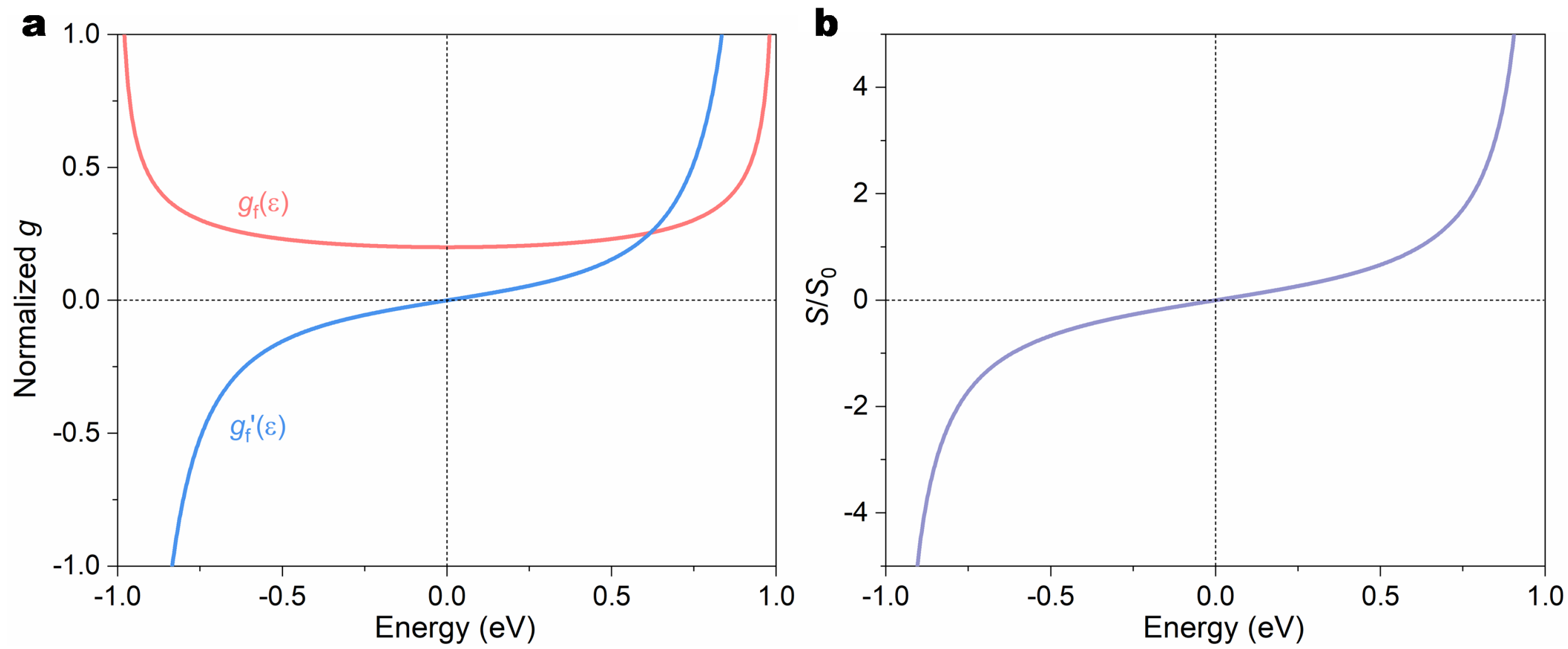


**Supplementary Fig. 2. Transport of flat band. a**, DOS $g_f(\varepsilon)$ and its derivative $g_f'(\varepsilon)$ for flat band. **b**, Seebeck response for flat band in unit of $S_0 = \pi^2\kappa_B^2T/3e$.

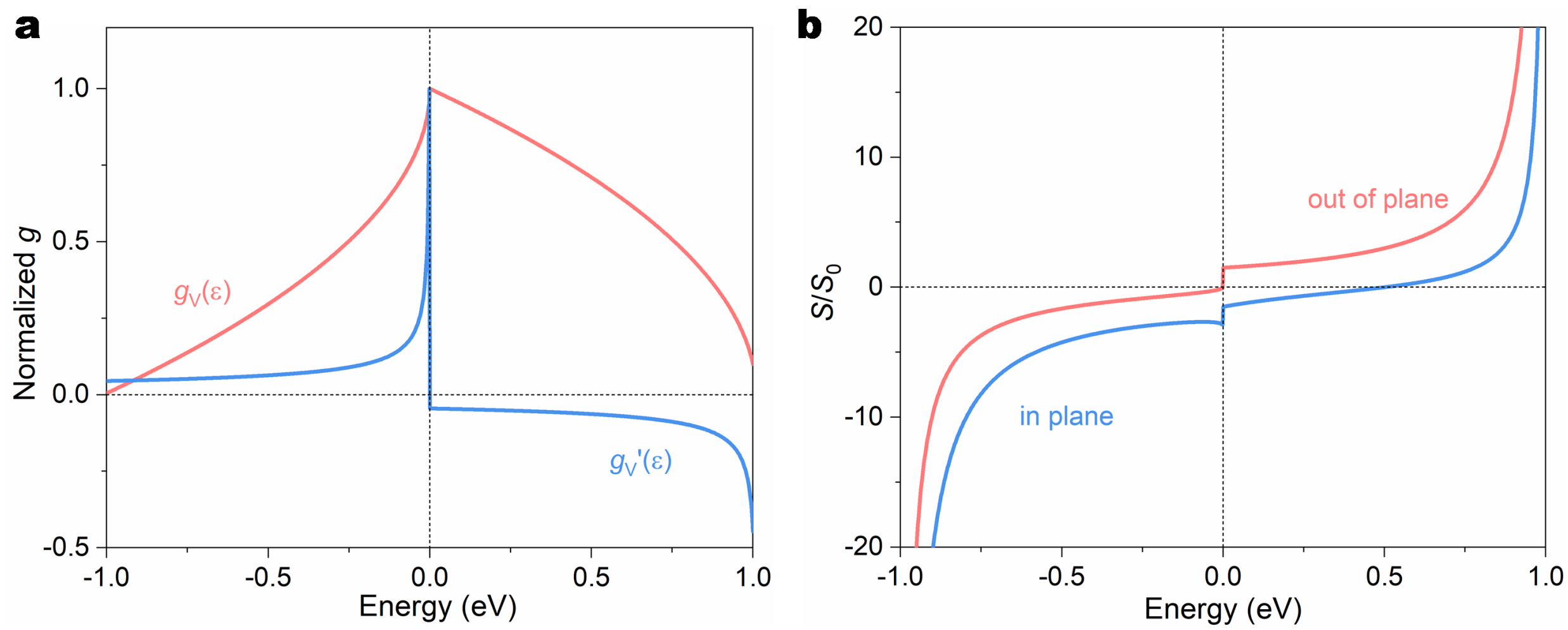


**Supplementary Fig. 3. Properties of VHS Hamiltonian. a**, DOS $g_V(\varepsilon)$ and its derivative $g_V'(\varepsilon)$. **b**, In-plane and out-of-plane Seebeck response in unit of $S_0 = \pi^2 \kappa_B^2 T/3e$.

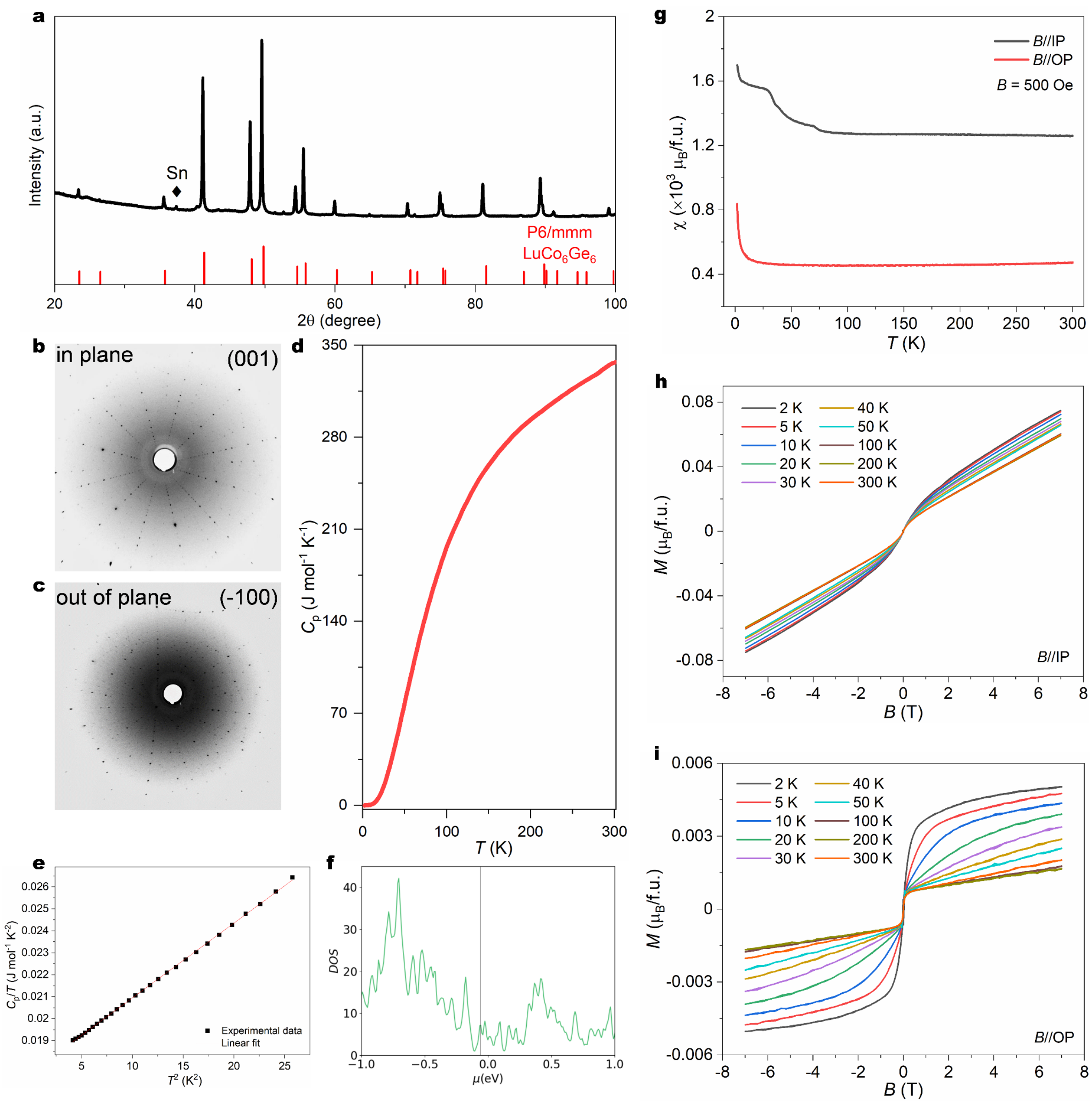


**Supplementary Fig. 4. Crystal structure, Laue, heat capacity and magnetism of $LuCo_6Ge_6$.** **a**, The powder x-ray diffraction (PXRD) pattern of $LuCo_6Ge_6$ single crystals. The diffraction pattern observed at room temperature aligns well with the P6/mmm space group, with only a minor impurity peak attributed to the Sn flux resulting from the grinding of multiple pieces for PXRD analysis. **b**,**c**, Laue diffraction patterns of in-plane (001) (**b**) and out-of-plane (-100) (**c**). **d**, Temperature dependence of heat capacity of $LuCo_6Ge_6$. **e**, Linear fitting of $T^2$ dependence of $C_p/T$ was used to estimate the electronic specific heat to be 0.01754 J $mol^{-1}\cdot K^{-2}$, corresponding to an experimental density of states of $g_{exp}$ = 7.44 states/eV. **f**, The calculated density of states for $LuCo_6Ge_6$ yielded a DOS of 7.05 states/eV at −60 meV, which is in good agreement with the experimental estimate. **g**, Temperature-dependent magnetic susceptibility at $B$ = 500 Oe for the field along the in-plane and out-of-plane directions. **h**,**i**, Field-dependent magnetization for the field along the in-plane (**h**) and out-of-plane directions (**i**).

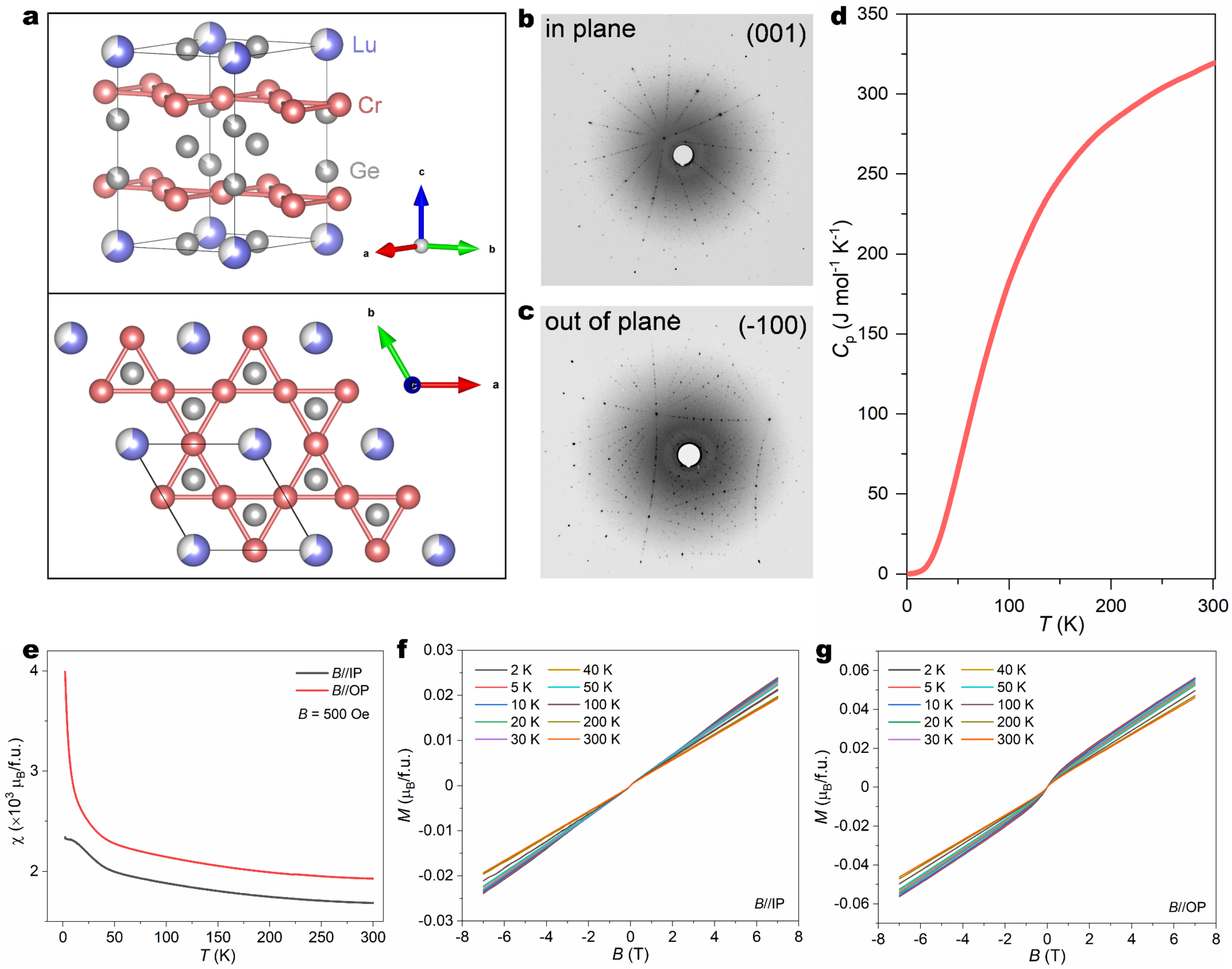


**Supplementary Fig. 5. Crystal structures, heat capacity and magnetism of $LuCr_6Ge_6$. a**, Crystal structure of $LuCr_6Ge_6$ kagome metal. **b**,**c**, Laue diffraction patterns of in-plane (001) (**b**) and out-of-plane (-100) (**c**). **d**, Temperature dependence of heat capacity of $LuCr_6Ge_6$. **e**, Temperature-dependent magnetic susceptibility at $B$ = 500 Oe for the field along the in-plane and out-of-plane directions. **f**,**g**, Field-dependent magnetization for the field along the in-plane (**f**) and out-of-plane directions (**g**).

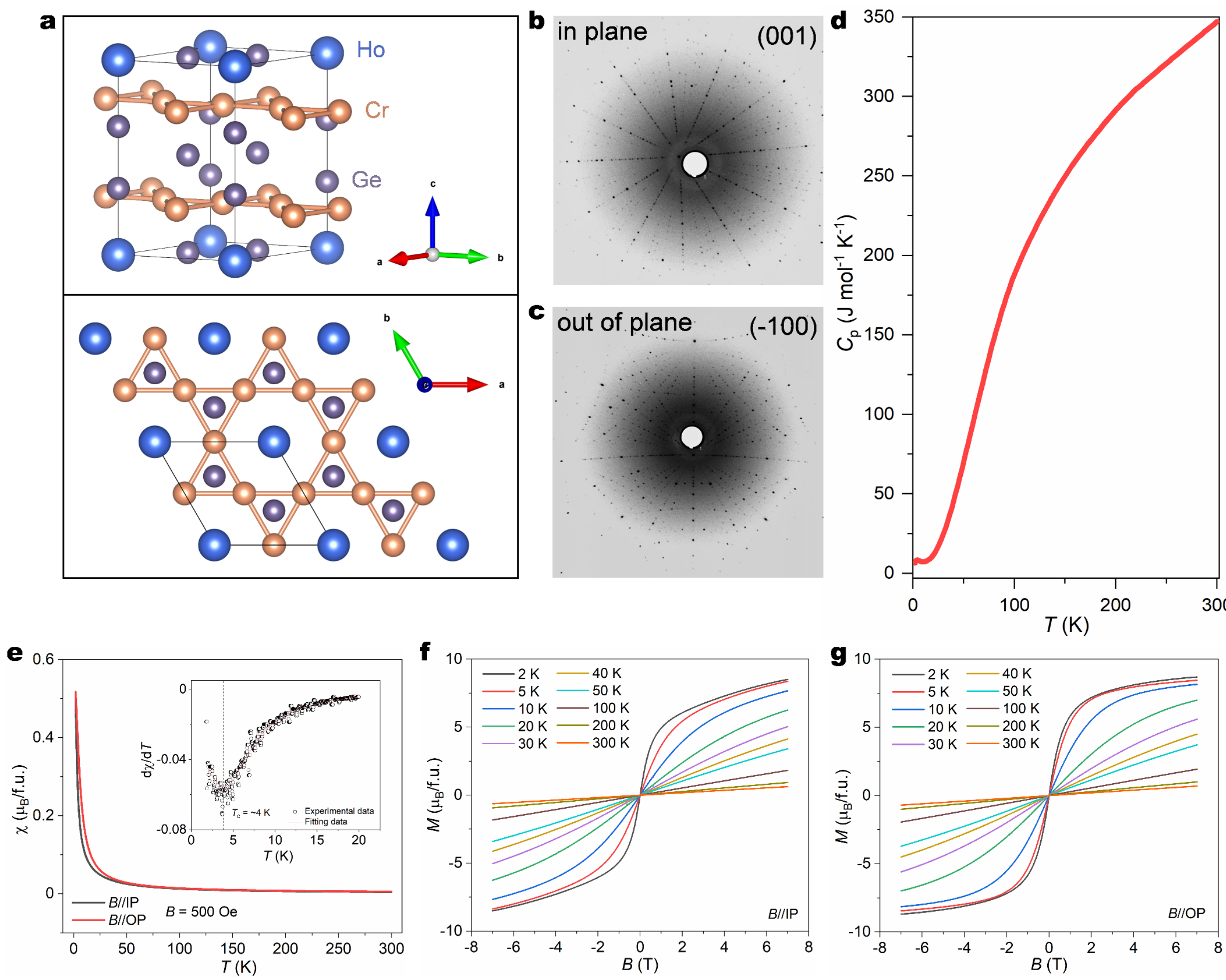


**Supplementary Fig. 6. Crystal structures, heat capacity and magnetism of $HoCr_6Ge_6$. a**, Crystal structure of $HoCr_6Ge_6$ kagome metal. **b**,**c**, Laue diffraction patterns of in-plane (001) (**b**) and out-of-plane (-100) (**c**). **d**, Temperature dependence of heat capacity of $HoCr_6Ge_6$. **e**, Temperature-dependent magnetic susceptibility at $B$ = 500 Oe for the field along the in-plane and out-of-plane directions. The inset displays the d$\chi$/d$T$ vs $T$ curve, revealing a magnetic transition temperature of ~4 K. **f**,**g**, Field-dependent magnetization for the field along the in-plane (**f**) and out-of-plane directions (**g**).

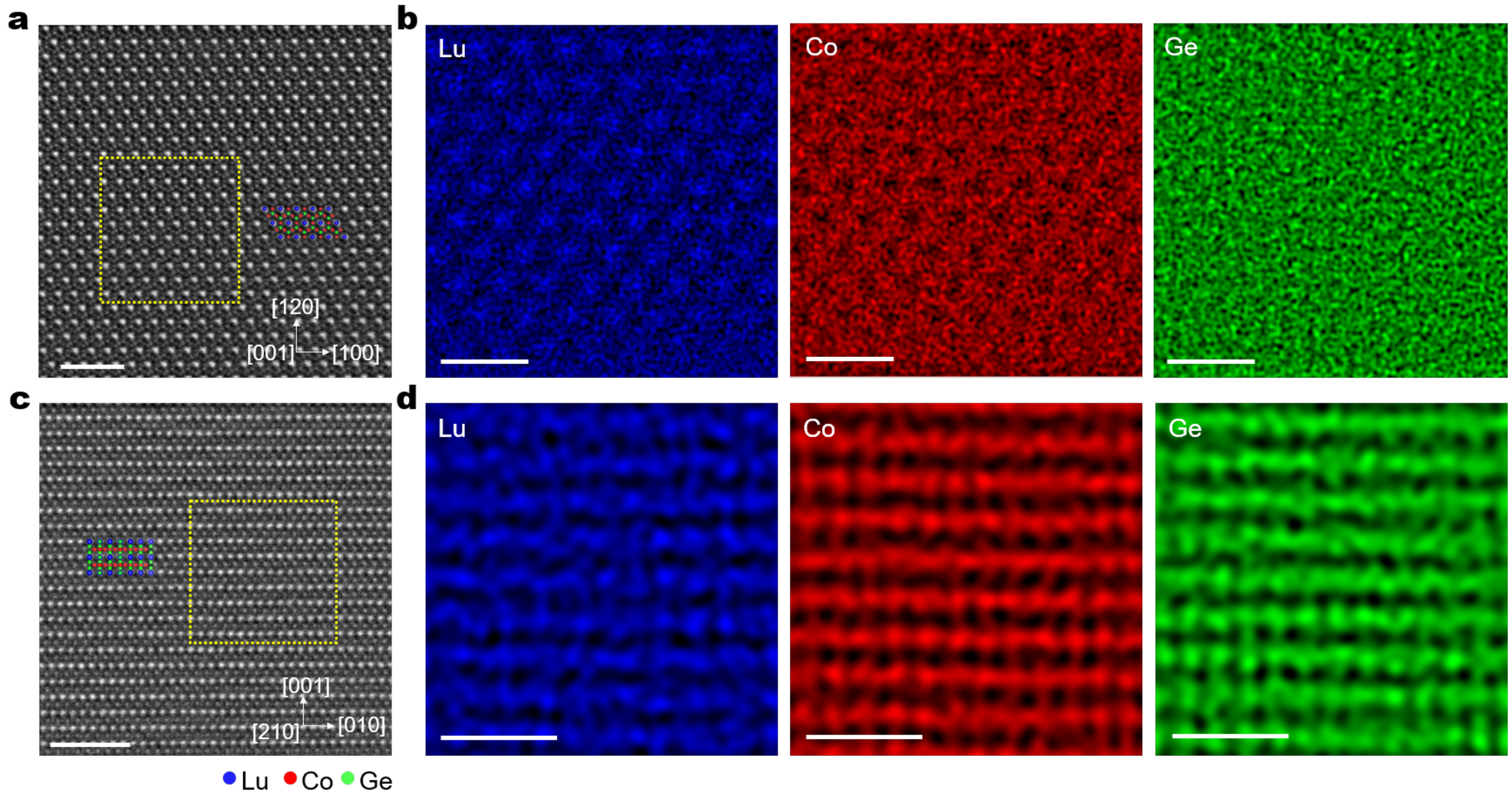


**Supplementary Fig. 7. HAADF-STEM images and EDS mappings of $LuCo_6Ge_6$. a**, HAADF-STEM image of $LuCo_6Ge_6$ along the [001] zone axis, overlaid with the projected atomic mode. Scale bar, 2 nm. **b**, EDS mappings corresponding to the yellow dashed box in (**a**). Scale bars, 1 nm. **c**, HAADF-STEM image of $LuCo_6Ge_6$ along the [210] zone axis, overlaid with the projected atomic mode. **d**, EDS mappings corresponding to the yellow dashed box in (**c**). Scale bars, 5 Å.

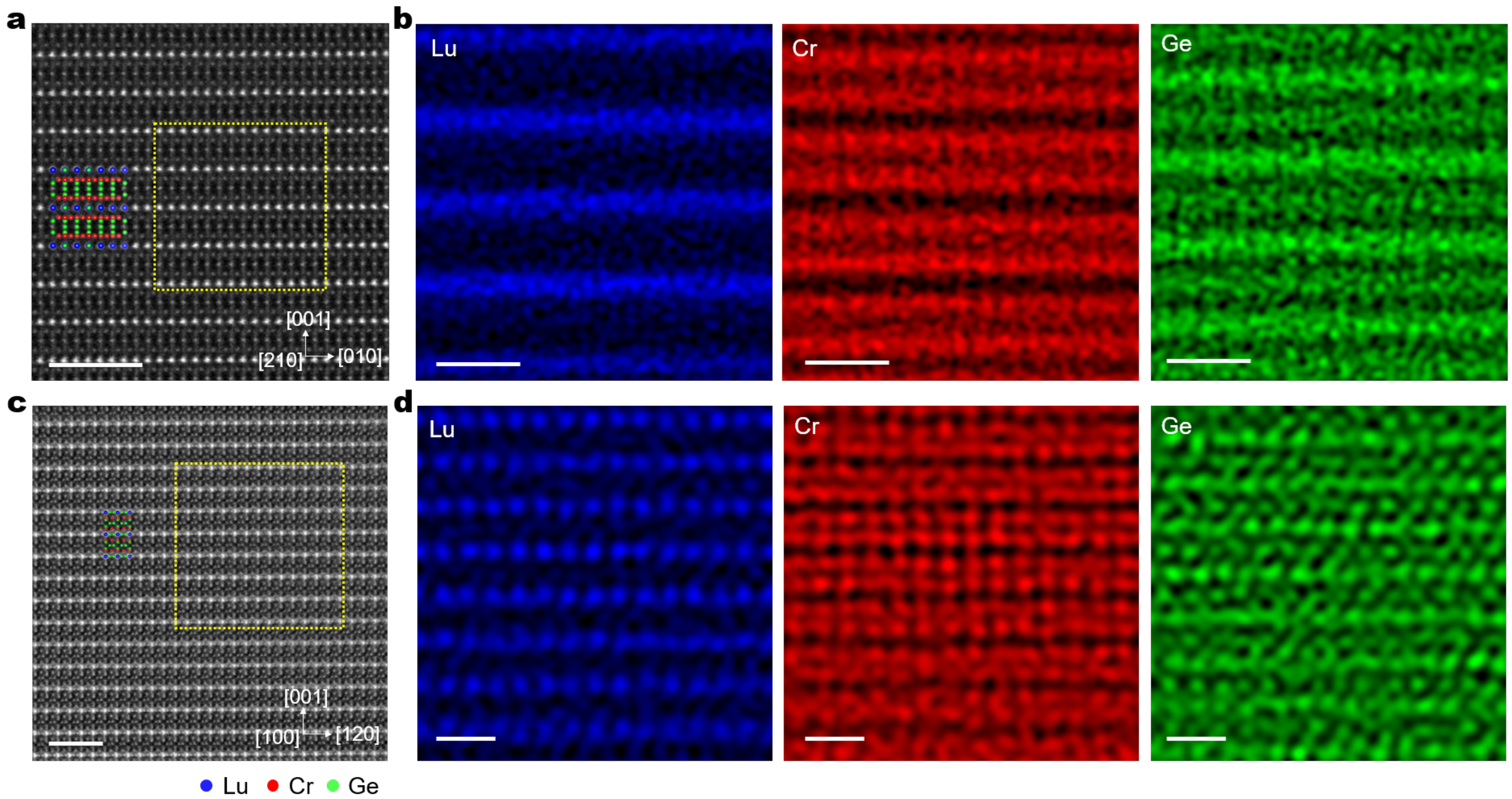


**Supplementary Fig. 8. HAADF-STEM images and corresponding EDS mappings of $LuCr_6Ge_6$. a**, HAADF-STEM image of $LuCr_6Ge_6$ along the [210] zone axis, overlaid with the projected atomic mode. Scale bar, 2 nm. **b**, EDS mappings corresponding to the yellow dashed box in (**a**). Scale bars, 1 nm. **c**, HAADF-STEM image of $LuCr_6Ge_6$ along the [100] zone axis, overlaid with the projected atomic mode. **d**, EDS mappings corresponding to the yellow dashed box in (**c**). Scale bars, 1 nm.

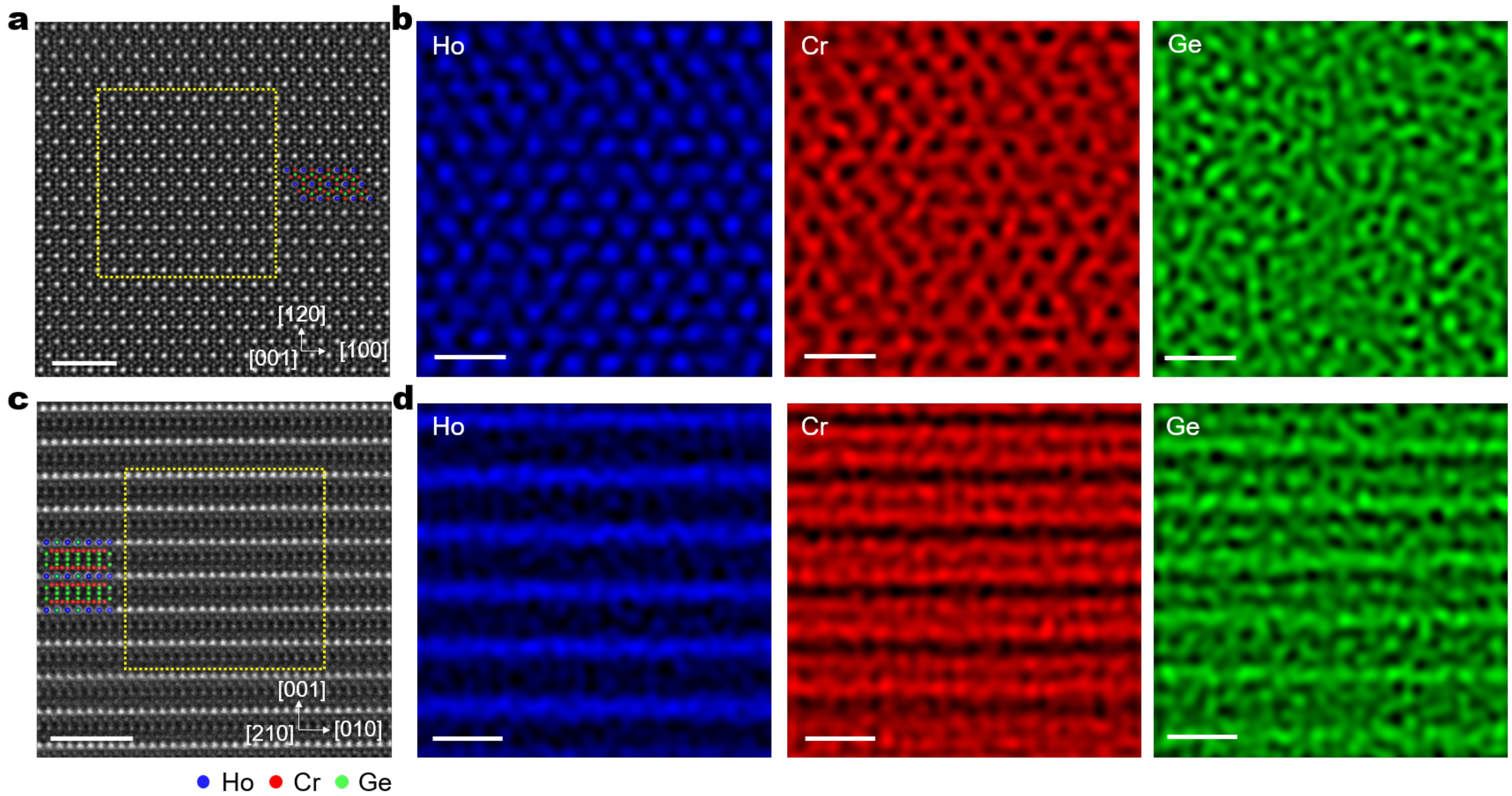


**Supplementary Fig. 9. HAADF-STEM images and corresponding EDS mappings of $HoCr_6Ge_6$.** **a**, HAADF-STEM image of $HoCr_6Ge_6$ along the [001] zone axis, overlaid with the projected atomic mode. Scale bar, 2 nm. **b**, EDS mappings corresponding to the yellow dashed box in (a). Scale bars, 1 nm. **c**, HAADF-STEM image of $HoCr_6Ge_6$ along the [210] zone axis, overlaid with the projected atomic mode. **d**, EDS mappings corresponding to the yellow dashed box in (**c**). Scale bars, 1 nm.

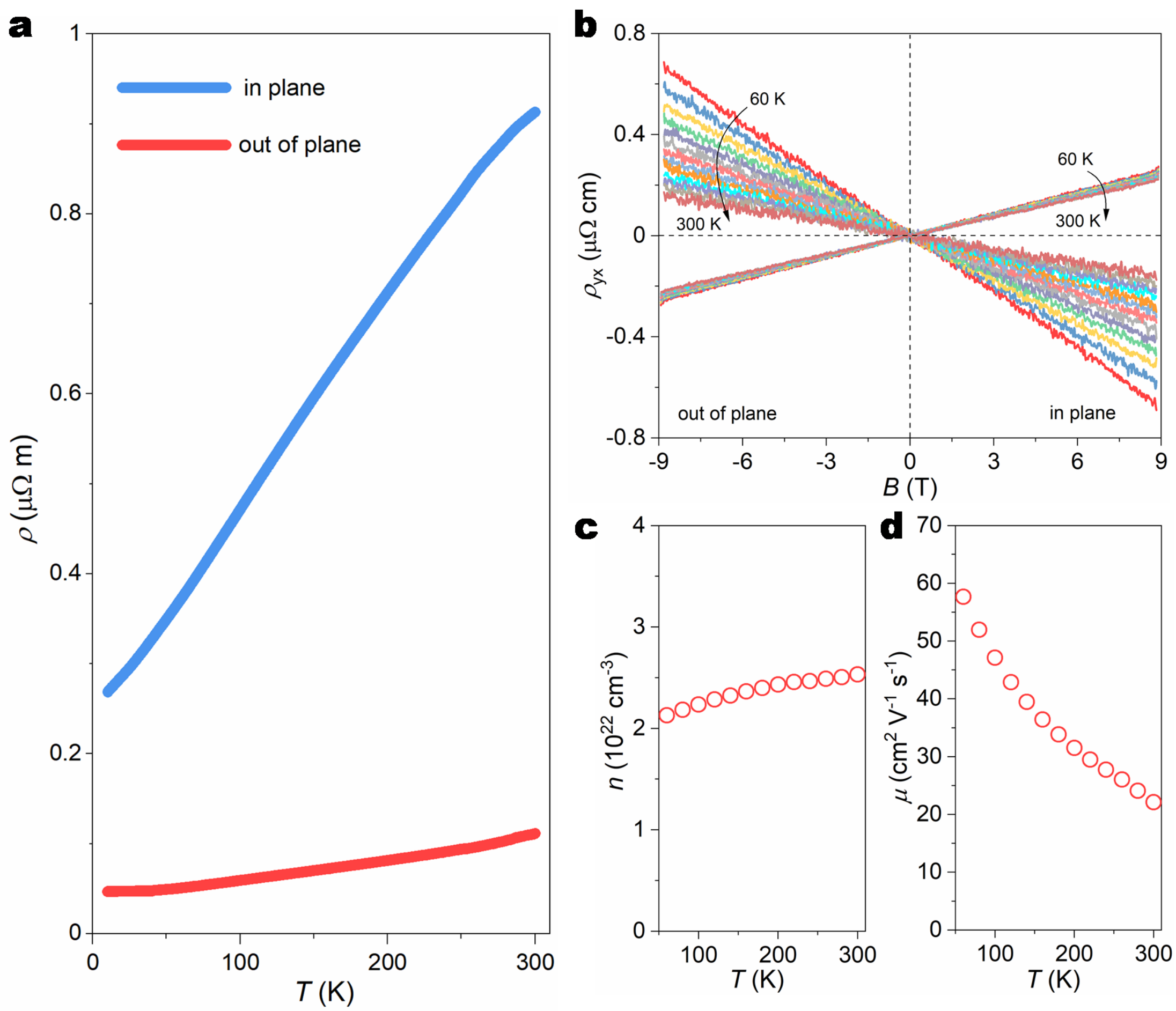


**Supplementary Fig. 10. Electrical transport properties of $LuCo_6Ge_6$ kagome metal. a**, Temperature dependence of resistivity $\rho$. **b**, Hall resistivity $\rho_{yx}$ as a function of the magnetic field. **c**, Carrier concentration. **d**, Carrier mobility.

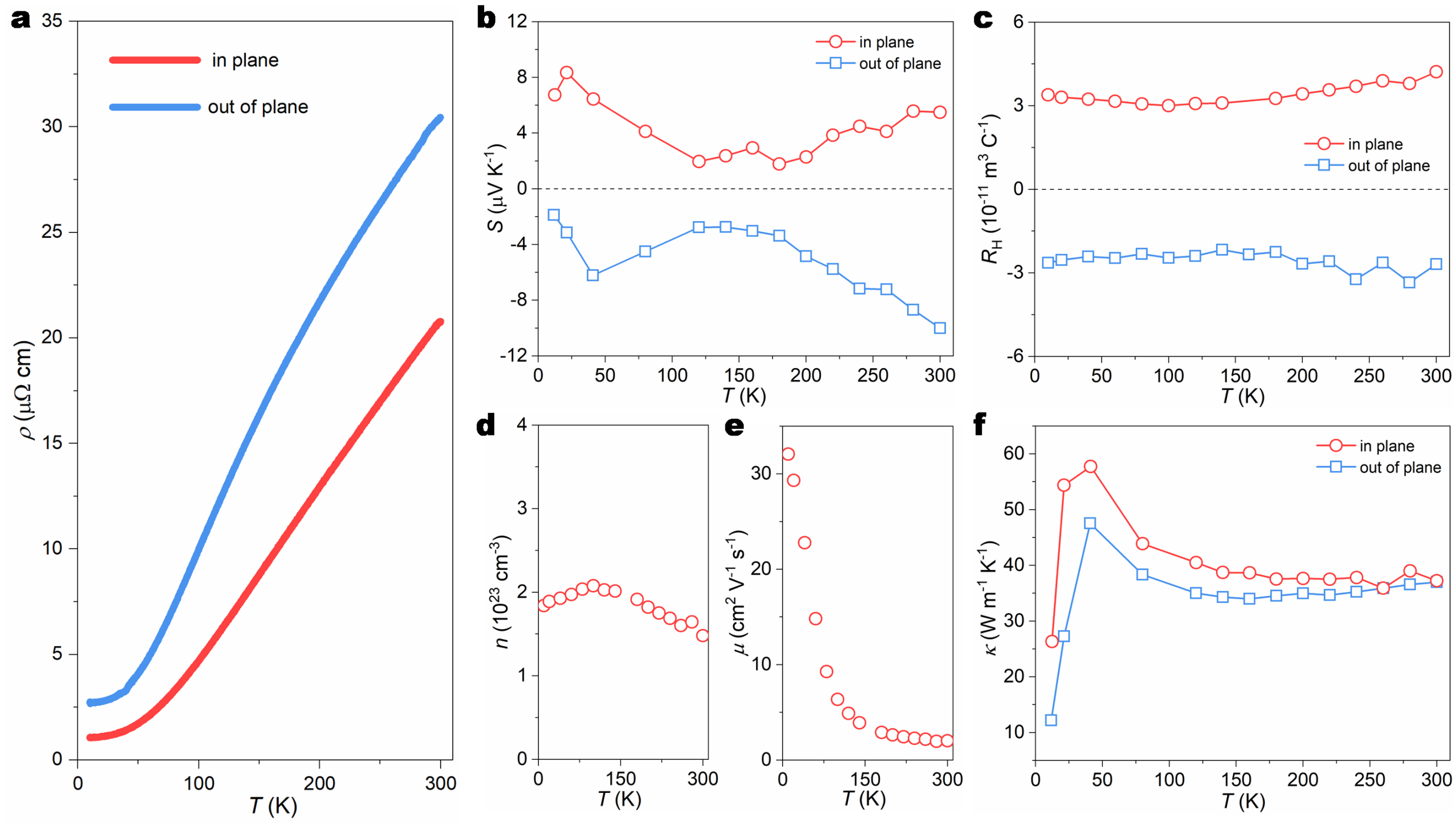


**Supplementary Fig. 11. Electrical transport properties of $LuCr_6Ge_6$ kagome metal. a**, Temperature dependence of resistivity $\rho$. **b**, Temperature-dependent in-plane ($S_{IP}$) and out-of-plane ($S_{OP}$) Seebeck coefficients. **c**, Hall coefficients ($R_H$) as a function of temperature. **d**, Carrier concentration. **e**, Carrier mobility. **f**, Temperature dependence of thermal conductivities.

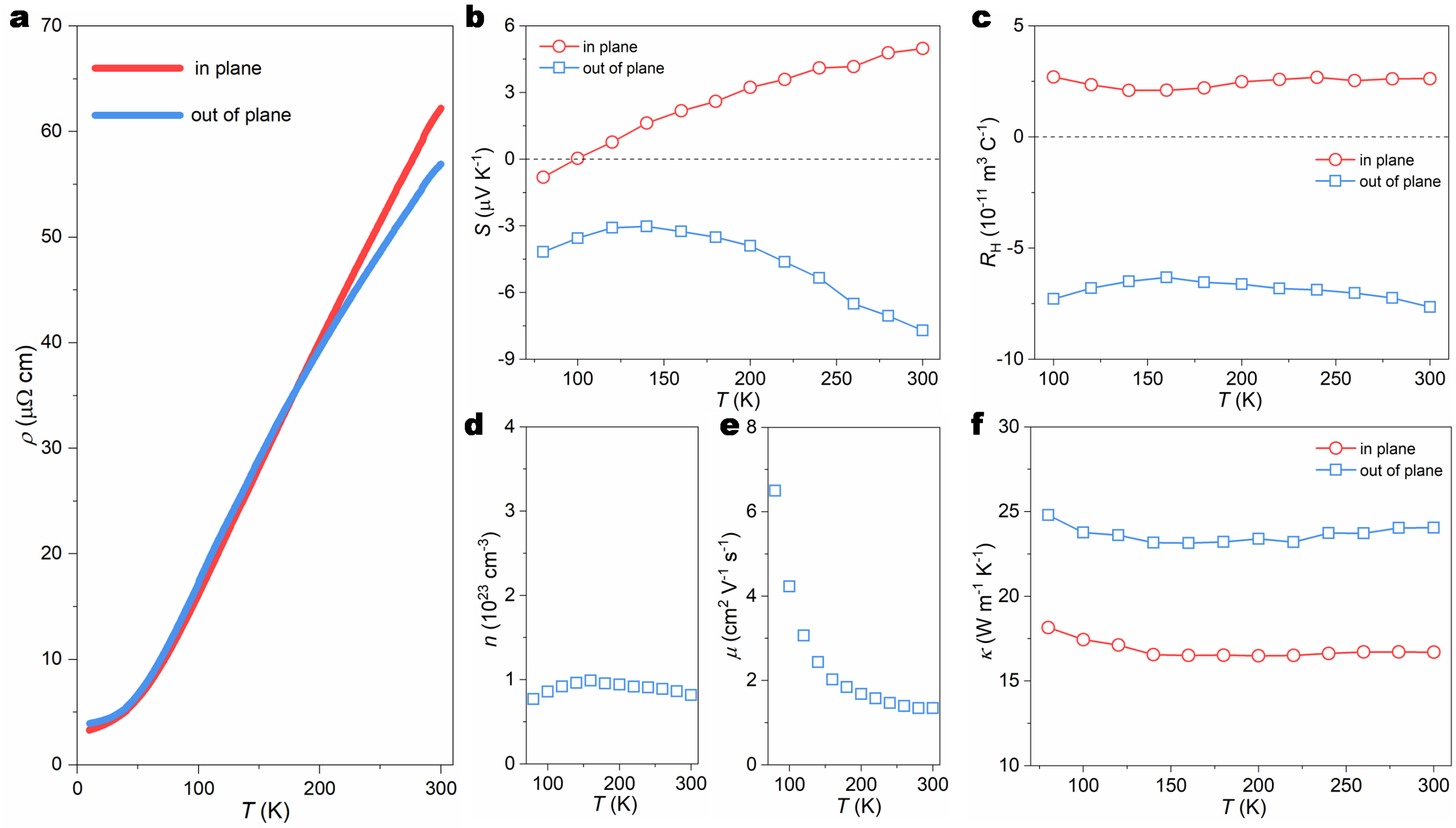


**Supplementary Fig. 12. Electrical transport properties of $HoCr_6Ge_6$ kagome metal. a**, Temperature dependence of resistivity $\rho$. **b**, Temperature-dependent in-plane ($S_{IP}$) and out-of-plane ($S_{OP}$) Seebeck coefficients. **c**, Hall coefficients ($R_H$) as a function of temperature. **d**, Carrier concentration. **e**, Carrier mobility. **f**, Temperature dependence of thermal conductivities.

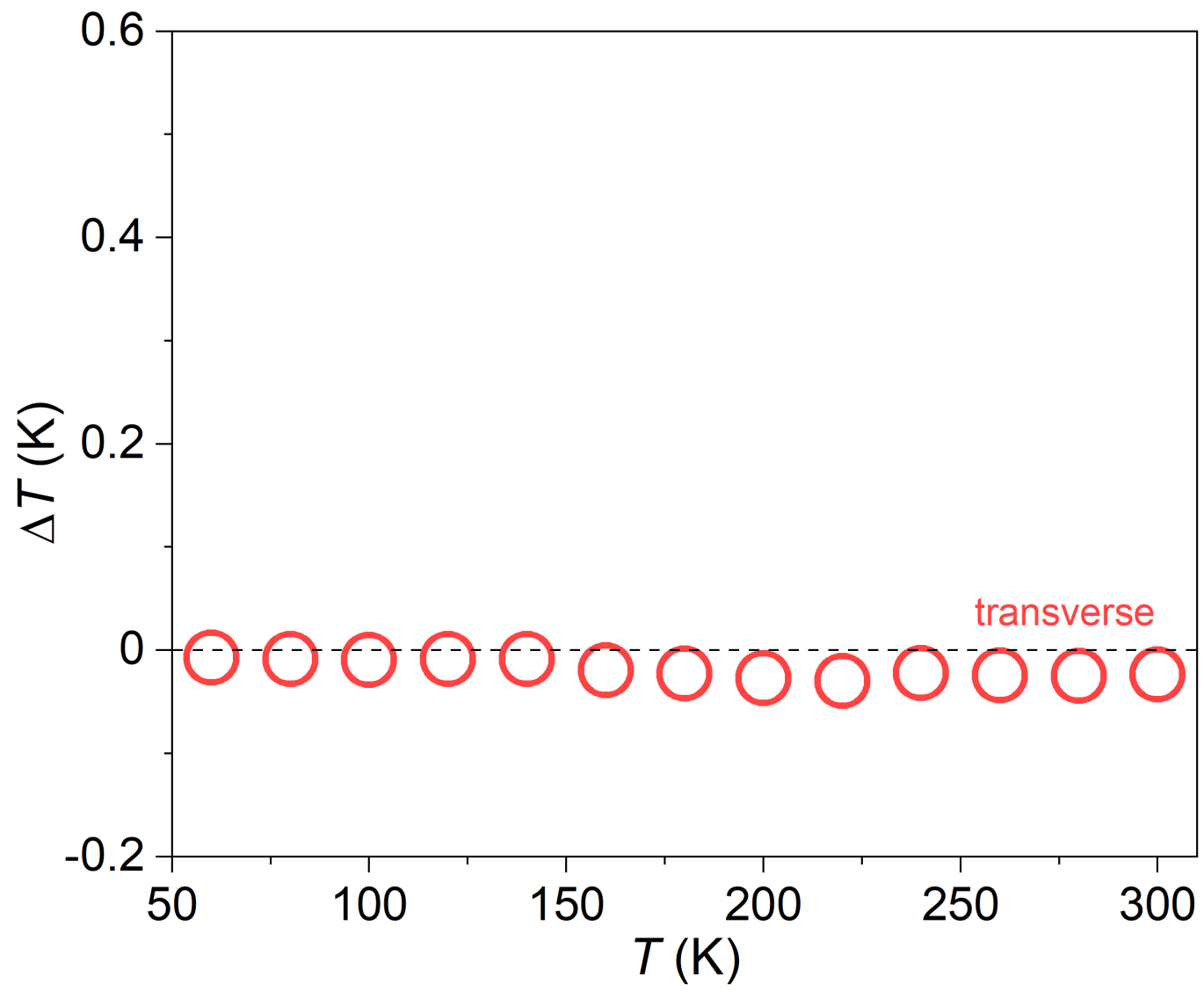


**Supplementary Fig. 13. Temperature difference.** Temperature-dependent transverse temperature differences of $LuCo_6Ge_6$ transverse measurement setup.

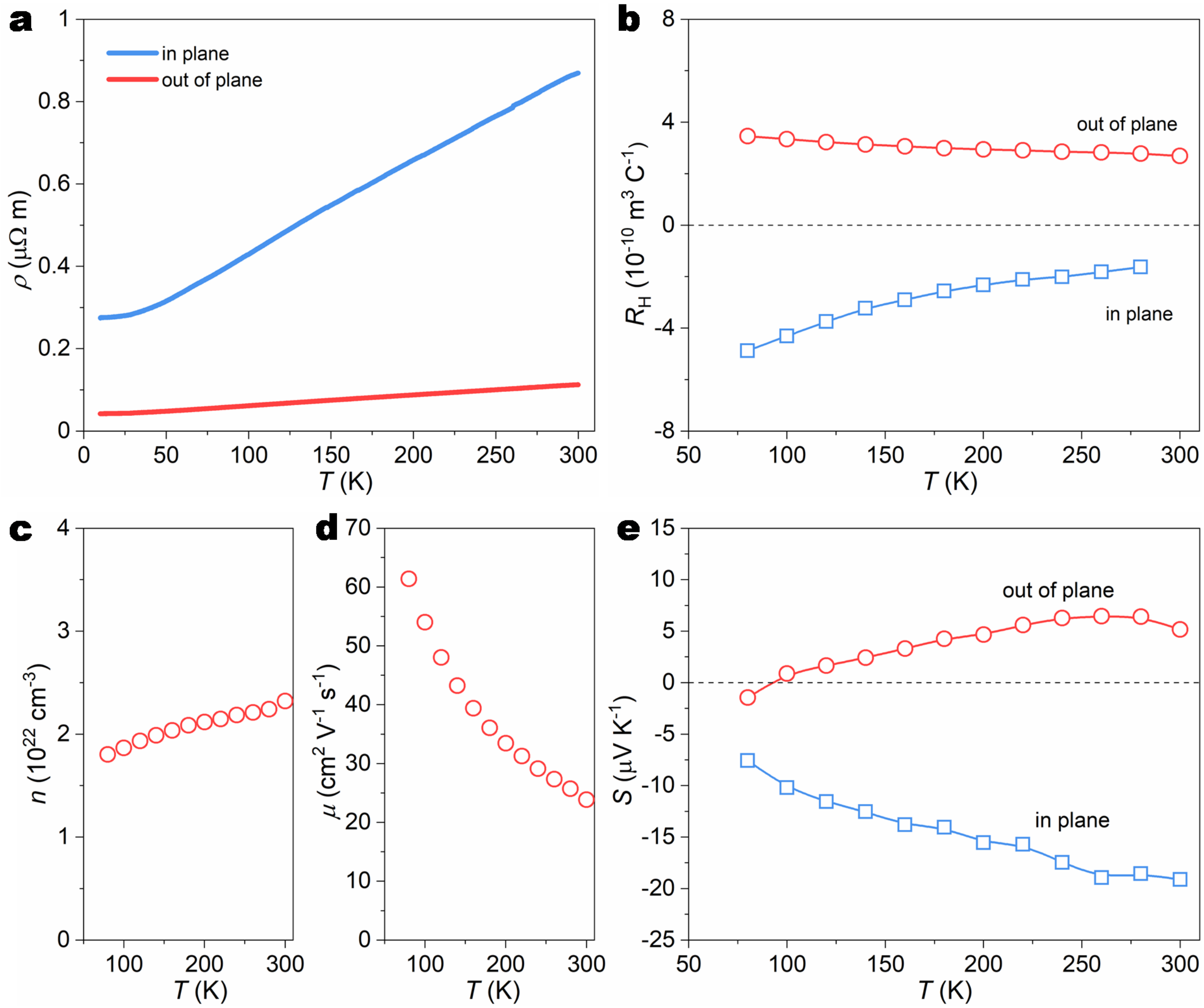


**Supplementary Fig. 14. Electrical properties of another batch of $LuCo_6Ge_6$. a**, Temperature dependence of resistivity $\rho$. **b**, Hall coefficients ($R_H$) as a function of temperature. **c**, Carrier concentration. **d**, Carrier mobility. **e**, Temperature-dependent in-plane ($S_{IP}$) and out-of-plane ($S_{OP}$) Seebeck coefficients.

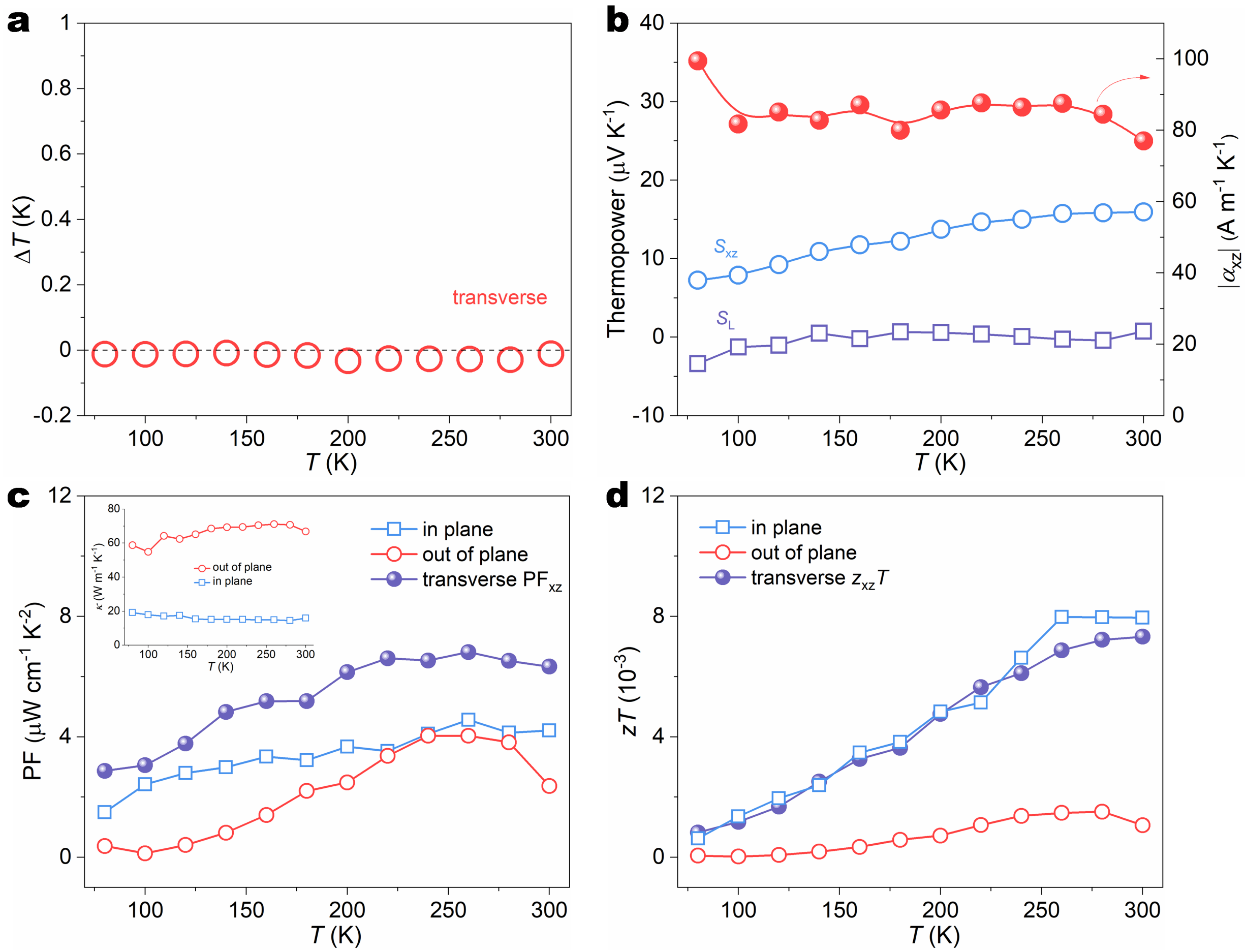


**Supplementary Fig. 15. Transverse thermoelectric performance of another batch of $LuCo_6Ge_6$.** **a**, Temperature-dependent transverse temperature differences. **b**, Longitudinal thermopowers $S_L$, transverse thermopower $S_{xz}$, and transverse Peltier conductivity $|\alpha_{xz}|$ versus temperature. **c**, Temperature dependence of power factors and thermal conductivities (inset). **d**, Thermoelectric figure of merit $zT$ as a function of temperature.

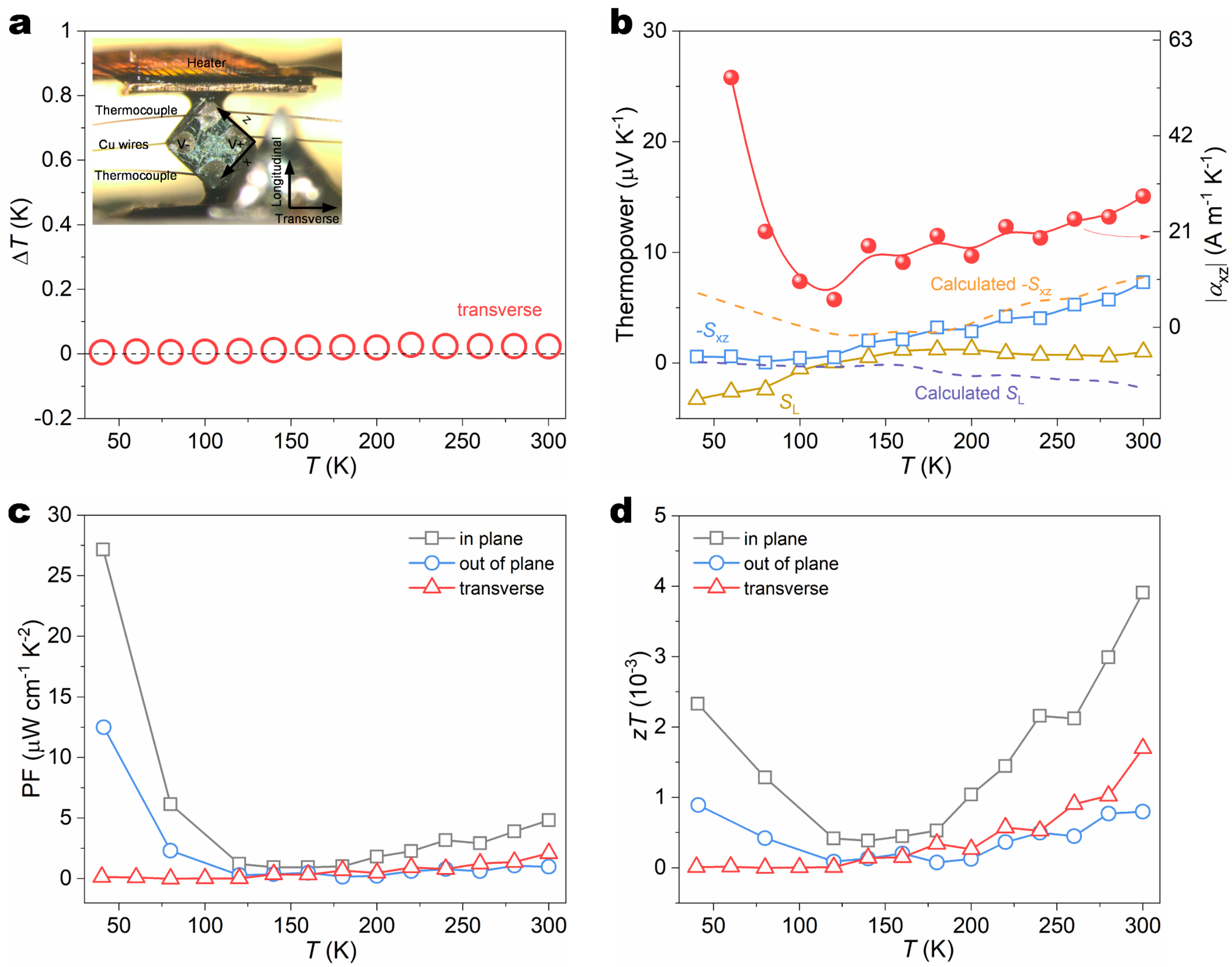


**Supplementary Fig. 16. Transverse thermoelectric performance of $LuCr_6Ge_6$ kagome metal.** **a**, Temperature-dependent transverse temperature differences. Inset: Experimental setup for transverse thermoelectric measurement. **b**, Longitudinal thermopowers $S_L$, transverse thermopower $S_{xz}$, and transverse Peltier conductivity $|\alpha_{xz}|$ versus temperature. Calculated $S_L$ and $S_{xz}$ are represent by purple and orange dashed lines, respectively. **c**, Power factors as a function of temperature. **d**, Temperature dependence of thermoelectric figure of merit $zT$.

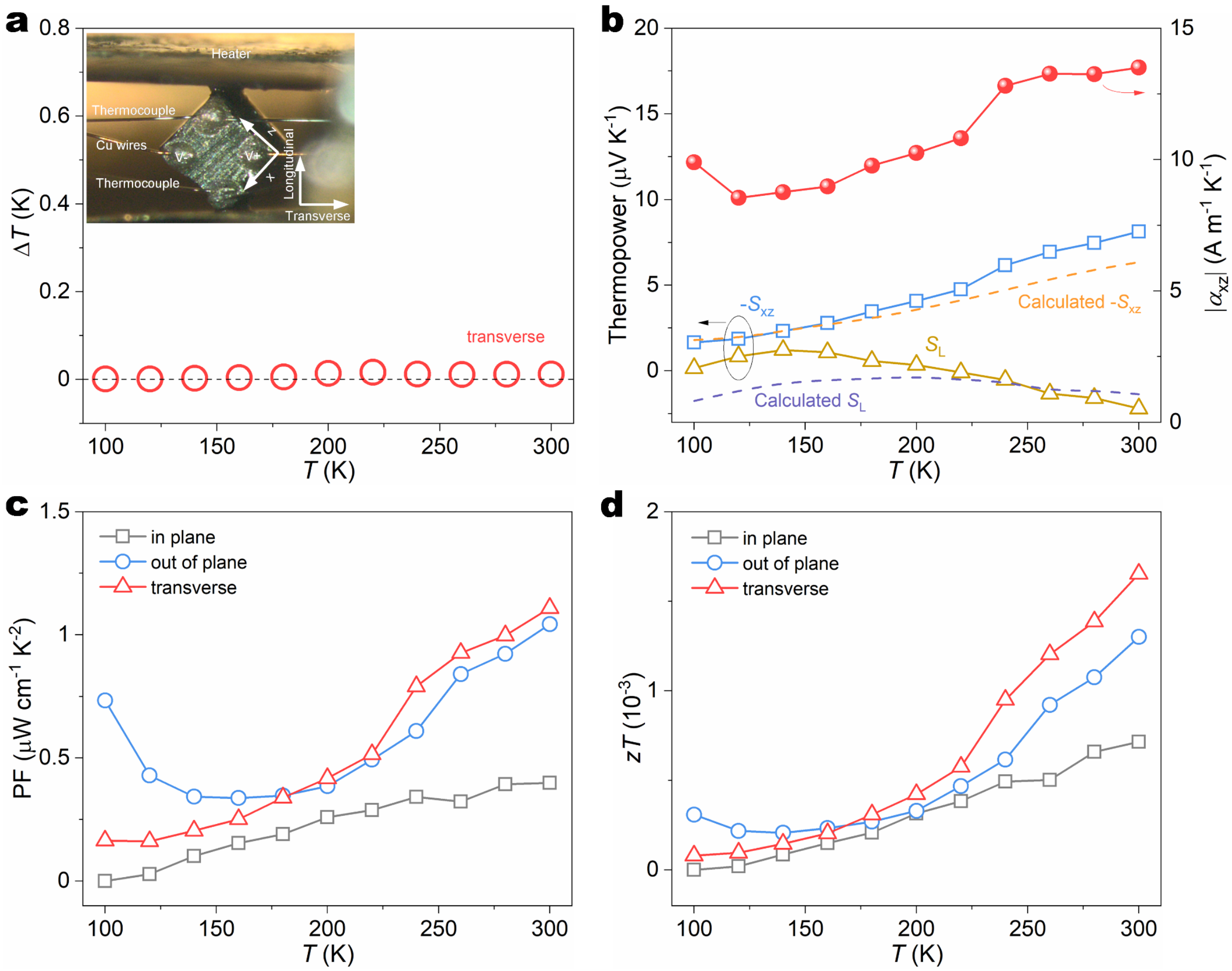


**Supplementary Fig. 17. Transverse thermoelectric performance of $HoCr_6Ge_6$ kagome metal.** **a**, Temperature-dependent longitudinal and transverse temperature differences. Inset: Experimental setup for transverse thermoelectric measurement. **b**, Longitudinal thermopowers $S_L$, transverse thermopower $S_{xz}$, and transverse Peltier conductivity $|\alpha_{xz}|$ versus temperature. Calculated $S_L$ and $S_{xz}$ are represented by purple and orange dashed lines, respectively. **c**, Power factors as a function of temperature. **d**, Temperature dependence of thermoelectric figure of merit $zT$.

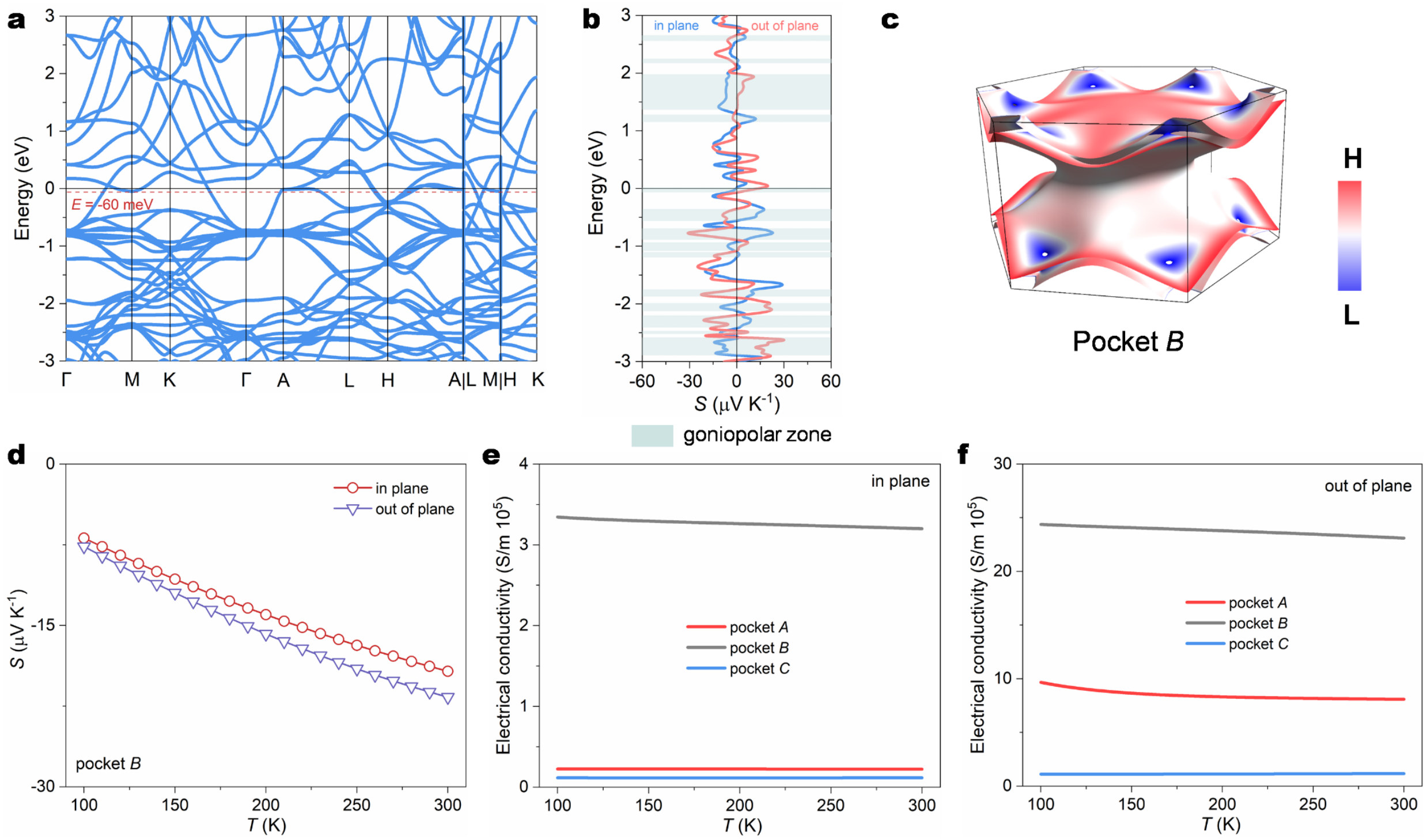


**Supplementary Fig. 18. Theoretical analysis of goniopolarity in $LuCo_6Ge_6$. a**, Band structure of $LuCo_6Ge_6$. The dashed line shows the real Fermi level. **b**, The energy-dependent Seebeck coefficients. **c**, The electron-dominated pocket *B*. **d**, Calculated Seebeck coefficients for pocket *B*. **e**,**f**, The in-plane (**e**) and (**f**) out-of-plane electrical conductivities of three pockets.

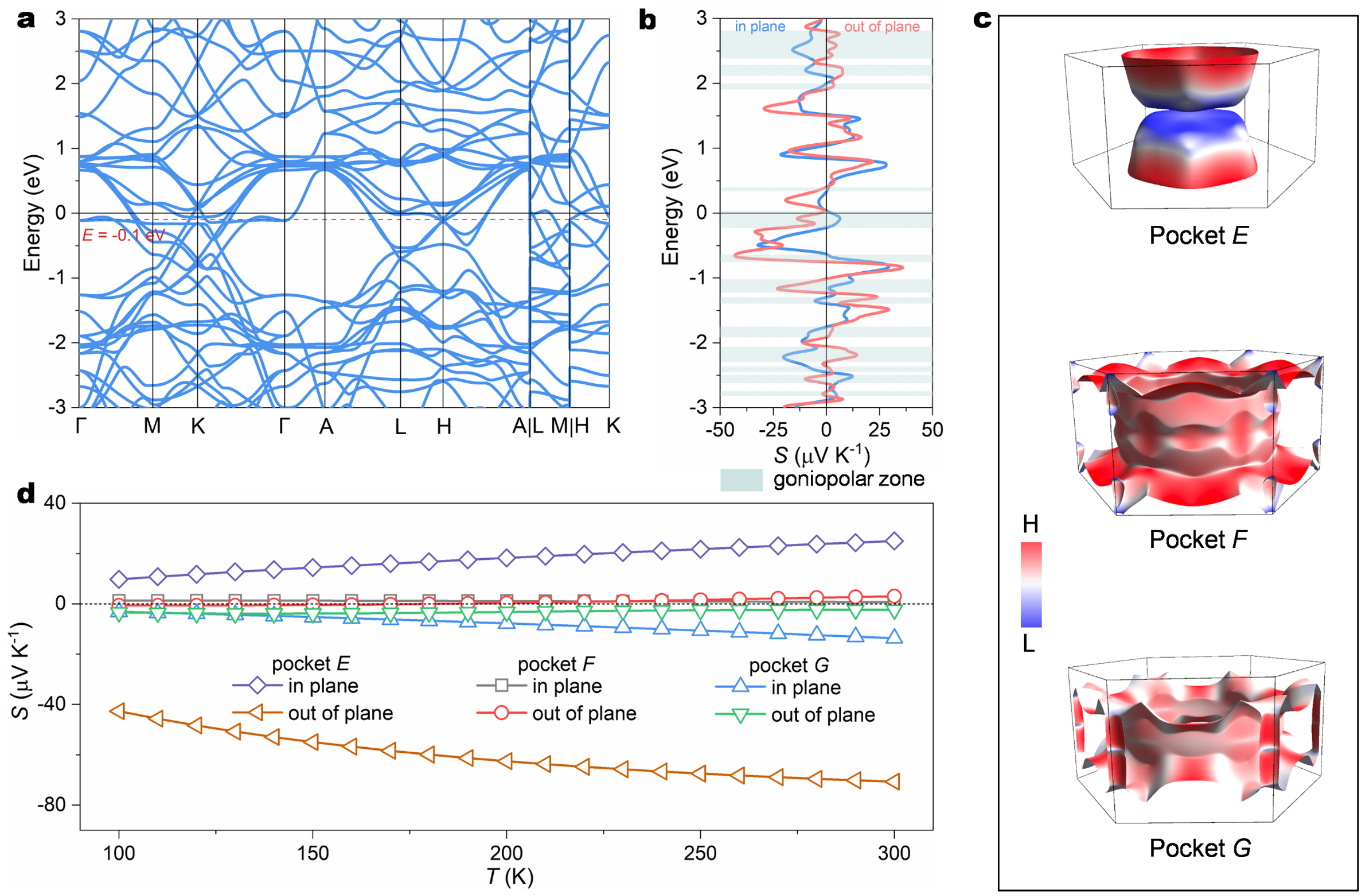


**Supplementary Fig. 19. Theoretical analysis of goniopolarity in $LuCr_6Ge_6$. a**, Band structure of $LuCr_6Ge_6$. The dashed line shows the real Fermi level. **b**, The energy-dependent Seebeck coefficients. **c**, Three Fermi pockets. **d**, Calculated Seebeck coefficients for each individual pocket in $LuCr_6Ge_6$.

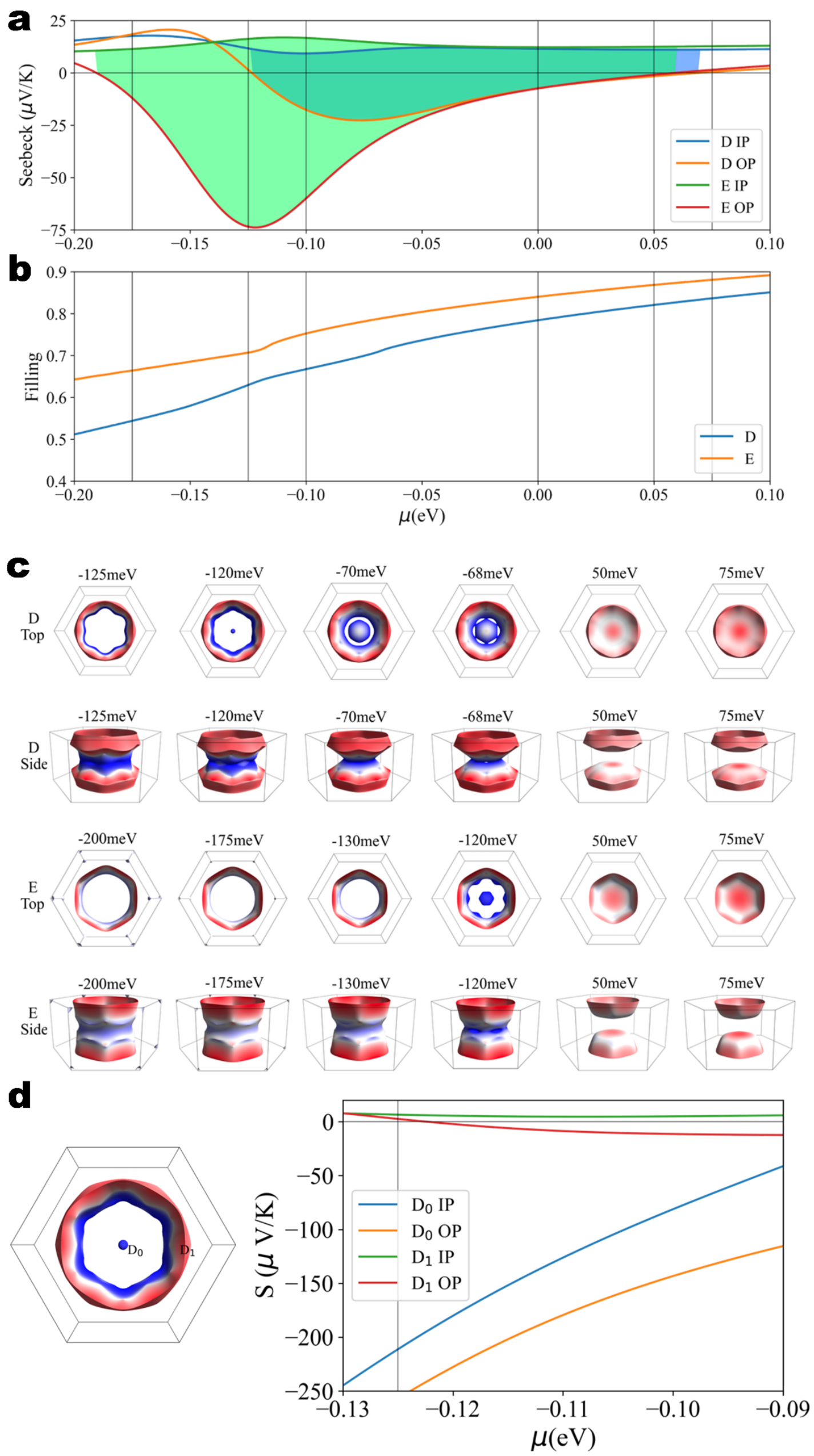


**Supplementary Fig. 20. Theoretical analysis of goniopolarity in $LuCr_6Ge_6$. a**,**b**, Band-resolved in-plane and out-of-plane Seebeck response (a), and (b) band filling for band D and E as a function of chemical potential, which shows a total filling over 0.5 in this region. Additional vertical lines are included at $E_F$ = −175, −125, 0, 50, and 75 meV for reference. **c**, Top and side view of Fermi surfaces for band D and E for varied chemical. The evolution of the Fermi surface indicates that no sharp topological change or reconnection occurs exactly at the onset or disappearance of goniopolarity. **d**, Pocket-resolved Seebeck response for band D near the onset of goniopolarity, which shows the larger open hyperbolic pocket $D_1$ dominates the observed goniopolarity while the inside smaller isotropic pocket $D_0$ presents electron-like behavior.

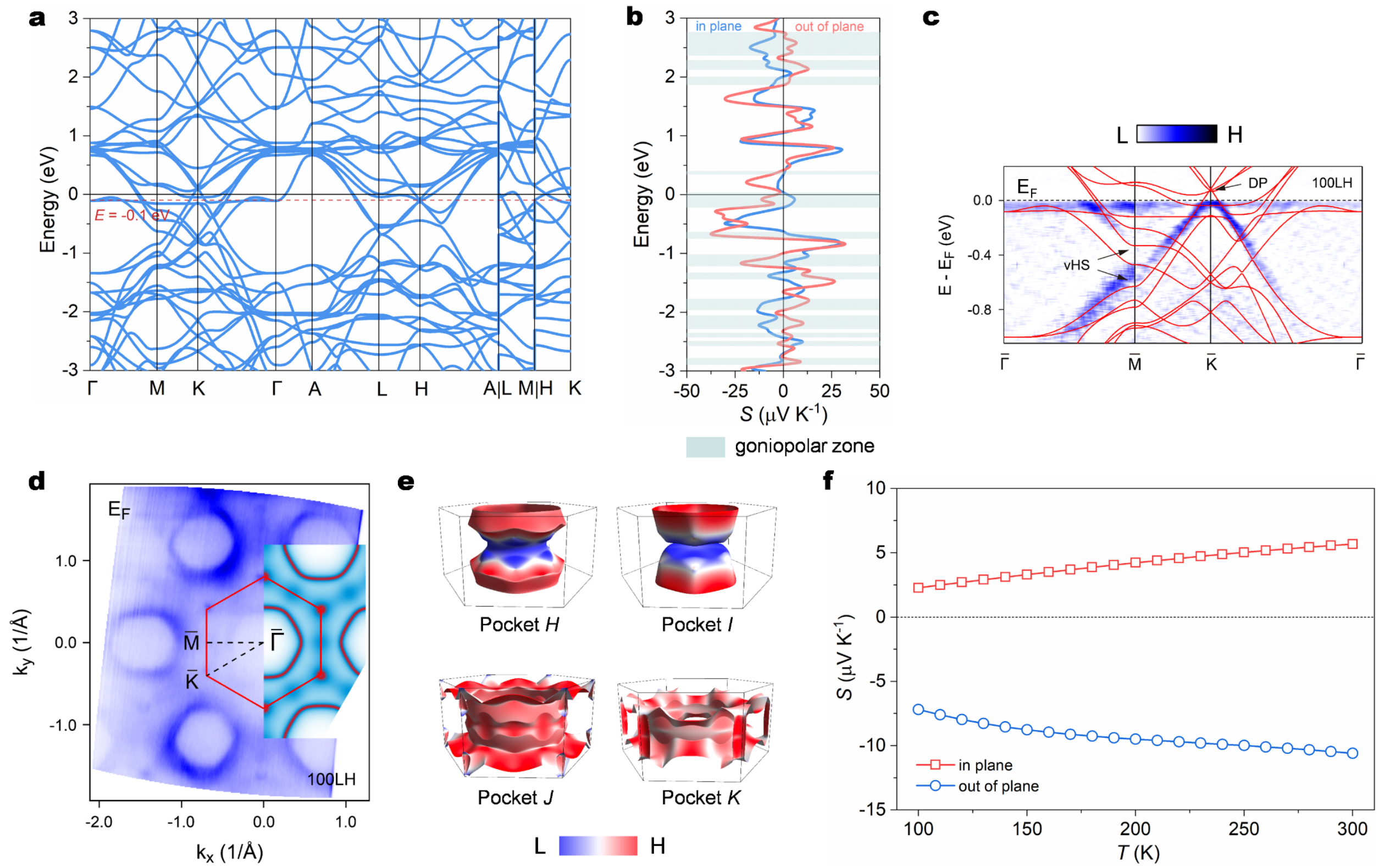


**Supplementary Fig. 21. Theoretical analysis of goniopolarity in $HoCr_6Ge_6$. a**, Band structure of $HoCr_6Ge_6$. The dashed line shows the real Fermi level. **b**, The energy-dependent Seebeck coefficients. **c**, Second derivative intensity plot of $HoCr_6Ge_6$ ($h\nu$ = 100 eV, LH polarization) recorded along the $\overline{\Gamma}$-$\overline{K}$-$\overline{M}$-$\overline{\Gamma}$ directions. The red solid curves are the DFT calculated bulk bands in $k_z = 0$ plane. The black arrows highlight the DP and vHSs observed in experiments, which are well reproduced by the calculations. By correlating the energy of the DP in $k_z = \pi$ plane with the experimental spectra, the real Fermi level was determined at $E_F = -0.1$ eV. **d**, Experimental and calculated Fermi surface mapping of $HoCr_6Ge_6$ ($T = 26$ K) taken by the 100-eV photons with linear horizontal (LH) polarization. The red solid curves indicate the surface Brillouin zone of (001) plane. **e**, Theoretical Fermi pockets (labeled as $H$, $I$, $J$, and $K$). **f**, The calculated temperature-dependent Seebeck coefficients.

**Supplementary Table 1.**
Summary of thermoelectric performance of our kagome metals at 300 K.

| Sample | $LuCo_6Ge_6$ | $LuCr_6Ge_6$ | $HoCr_6Ge_6$ |
|---|---|---|---|
| $\rho_{IP}$ (μΩ cm) | 91.2 | 20.8 | 62.2 |
| $\rho_{OP}$ (μΩ cm) | 11.1 | 30.5 | 56.9 |
| $n$ (×$10^{22}$ cm$^{-3}$) | 2.5 | 15 | 8 |
| $\mu$ (cm$^2$/Vs) | 22.1 | 2.0 | 1.3 |
| $S_{IP}$ (μV K$^{-1}$) | −17.2 | 5.5 | 5.0 |
| $S_{OP}$ (μV K$^{-1}$) | 9.1 | −10.0 | −7.7 |
| $PF_{IP}$ (μW cm$^{-1}$ K$^{-2}$) | 3.2 | 1.0 | 0.4 |
| $PF_{OP}$ (μW cm$^{-1}$ K$^{-2}$) | 7.4 | 4.8 | 1.0 |
| $\kappa_{IP}$ (W m$^{-1}$ K$^{-1}$) | 11.0 | 37.2 | 16.7 |
| $\kappa_{OP}$ (W m$^{-1}$ K$^{-1}$) | 69.3 | 37.0 | 24.1 |
| $z_{IP}T$ ($10^{-3}$) | 8.8 | 0.8 | 0.7 |
| $z_{OP}T$ ($10^{-3}$) | 3.2 | 3.9 | 1.3 |
| $S_L$ (μV K$^{-1}$) | −3.2 | 1.0 | −2.2 |
| $S_{xz}$ (μV K$^{-1}$) | 18.4 | 7.3 | 8.1 |
| $\|\alpha_{xz}\|$ | 105.4 | 30.3 | 13.8 |
| $PF_{xz}$ (μW cm$^{-1}$ K$^{-2}$) | 8.2 | 2.1 | 1.1 |
| $z_{xz}T$ ($10^{-3}$) | 11.5 | 1.7 | 1.7 |
